# Nocturnal Intraocular Pressure Monitoring With a Soft Contact Lens Sensor for Glaucoma Management

**DOI:** 10.1002/adma.73813

**Published:** 2026-06-23

**Authors:** Yumin Dai, Tristan Michael Long, Oluwabunmi T. Oladele, Youngoh Lee, Feiyang Li, Ziheng Wang, Yeonji Oh, Junsang Lee, Taewoong Park, Tianhao Yu, Seokkyoon Hong, Kyeonghee Lim, Jinheon Jeong, Dawn Meyer Schneider, Hyerim Ra, Bryan W. Boudouris, Gillian C. Shaw, Shin Ae Park, Pete S. Kollbaum, Chi Hwan Lee

**Affiliations:** ^1^ School of Materials Engineering Purdue University West Lafayette IN USA; ^2^ School of Optometry Indiana University Bloomington IN USA; ^3^ Department of Veterinary Clinical Sciences Purdue University West Lafayette IN USA; ^4^ Weldon School of Biomedical Engineering Purdue University West Lafayette IN USA; ^5^ School of Mechanical Engineering Purdue University West Lafayette IN USA; ^6^ Charles D. Davidson School of Chemical Engineering Purdue University West Lafayette IN USA; ^7^ Department of Chemistry Purdue University West Lafayette IN USA; ^8^ School of Veterinary Medicine University of Wisconsin‐Madison Madison WI USA; ^9^ Elmore Family School of Electrical and Computer Engineering Purdue University West Lafayette IN USA; ^10^ Center for Implantable Devices Purdue University West Lafayette IN USA

**Keywords:** glaucoma management, intraocular pressure monitoring, nocturnal monitoring, smart contact lenses

## Abstract

Measurement of nocturnal intraocular pressure (IOP) is essential for glaucoma management, yet recumbent IOP fluctuations during sleep remain largely unmeasured outside the clinic. Although existing eye‐worn wearable sensors enable noninvasive IOP monitoring, limitations in sensitivity, closed‐eye performance, and overnight wearability restrict accurate assessment of nocturnal IOP changes under sleep conditions. Here, we present a soft contact lens sensor designed for continuous monitoring of nocturnal IOP in a home setting. The sensor integrates a compliant conductor–based inductive–capacitive circuit directly onto a commercial soft contact lens, enabling close conformity to the eye. This design achieves the highest IOP sensitivity reported to date among wearable soft contact lens sensors, representing a substantial improvement over our earlier designs. As a result, the sensor provides stable and accurate IOP measurements under recumbent, closed‐eye sleep conditions. Animal studies in rabbits and dogs and pilot human measurements in healthy and glaucomatous eyes support preliminary ocular tolerability and feasible sensor performance, enabling posture‐dependent IOP tracking and detection of sleep‐relevant pressure elevations under the tested conditions. These results address key limitations of earlier contact lens sensors and support at‐home monitoring of nocturnal IOP dynamics.

## Graphical abstract

A soft contact lens sensor enables continuous, noninvasive monitoring of nocturnal intraocular pressure (IOP) under closed‐eye sleep conditions. By integrating a highly compliant inductive–capacitive circuit onto a commercial lens, the device achieves record sensitivity and stable performance. Animal and human studies demonstrate safety and reliable tracking, enabling practical at‐home assessment of nocturnal IOP dynamics for glaucoma management.

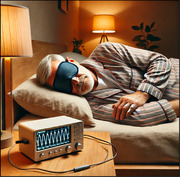

## 1 Introduction

Glaucoma, often referred to as the silent thief of sight, is the leading cause of irreversible blindness worldwide [1]. Epidemiological estimates indicate that more than 76 million people are affected globally, and this number is projected to reach 112 million by 2040 [2, 3]. The disease is characterized by progressive optic nerve degeneration driven by elevated intraocular pressure (IOP) resulting from impaired aqueous humor outflow [4, 5]. Although lowering IOP is the only proven method to slow progression, IOP is highly dynamic and influenced by circadian rhythm, posture, and daily activity [6]. Importantly, IOP often rises at night during sleep and recumbent conditions—precisely when direct measurement is not feasible in routine clinical care [7]. These nocturnal elevations are closely linked to accelerated nerve damage yet remain largely unmonitored by conventional tonometry, including Goldmann applanation, rebound, and dynamic contour tonometers, which provide only isolated IOP measurements [8].

Implantable sensors have been explored to address this limitation, but they require invasive surgery and raise long‐term safety concerns [9, 10, 11, 12]. Eye‐worn wearable systems—such as contact lens sensors including the Triggerfish lens—offer a noninvasive alternative for continuous IOP assessment [13, 14, 15, 16, 17, 18, 19]. However, these devices often rely on rigid microchips or piezoresistive pressure sensors in non‐commercial lenses, which can distort corneal curvature, limit sensitivity, reduce comfort, and introduce thermal load that compromises signal stability particularly under closed‐eye conditions [20]. Users frequently report foreign‐body sensation and ocular irritation during prolonged wear, making them particularly unsuitable for sleep‐time monitoring [21]. Although several previously reported contact lens sensors demonstrate wireless ocular pressure sensing, they provide only limited evidence of reliable performance under natural closed‐eye, nocturnal, or overnight monitoring conditions. As a result, current platforms remain limited in enabling noninvasive, comfortable, continuous monitoring of closed‐eye, supine, and nocturnal IOP dynamics.

Our earlier design—a soft contact lens sensor based on printed metal (e.g., Ag)–polymer composites—demonstrated comfortable prolonged wear and established the feasibility of continuous IOP monitoring using commercial hydrogel lenses [22]. By eliminating rigid microchips and integrating the sensing circuit directly onto standard soft lenses, this platform improved user comfort and mitigated several safety concerns associated with traditional electronics. However, its maximum sensitivity of 0.27 MHz mmHg^−1^ (1 121 ppm mmHg^−1^) limited its ability to resolve subtle nocturnal IOP fluctuations [22]. This challenge becomes particularly evident when lying down with eyes closed under natural sleep conditions, where eyelid and periocular tissue loading attenuate wireless coupling, corneal deformation amplitudes are minimal, and involuntary motions introduce additional signal instability [23]. Accurately detecting these subtle biomechanical changes requires substantially higher sensitivity and signal stability than what existing contact lens sensors can offer.

Here, we introduce a soft contact lens sensor engineered for continuous and reliable monitoring of nocturnal IOP dynamics in a home setting. The device integrates a soft, stretchable inductive–capacitive (LC) resonant circuit using liquid metal (LM) onto a commercial hydrogel contact lens under ambient processing conditions, enabling passive wireless sensing with negligible temperature increase. In contrast to prior wearable ocular tonometers, our platform is specifically developed for closed‐eye, recumbent, and nocturnal monitoring. Its direct integration onto a commercial soft contact lens further offers translational advantages for practical real‐world use. Table S1 compares the present system (1.08 MHz mmHg^−1^ or 2 542 ppm mmHg^−1^) with prior reports in terms of device characteristics and demonstrated monitoring scenarios [2, 22, 24, 25, 26, 27, 28]. Across ex vivo, animal, and human studies—including both healthy individuals and glaucoma patients—we demonstrate stable, physiologically relevant IOP readouts that extend from daytime activity into conditions representative of natural sleep. These results support the safety, comfort, and growing clinical promise of this platform, marking a meaningful step toward practical, at‐home monitoring of nocturnal IOP dynamics essential for glaucoma management.

## 2 Results

### 2.1 Soft Contact Lens Sensor Design for Continuous Nocturnal IOP Monitoring

**FIGURE 1:**
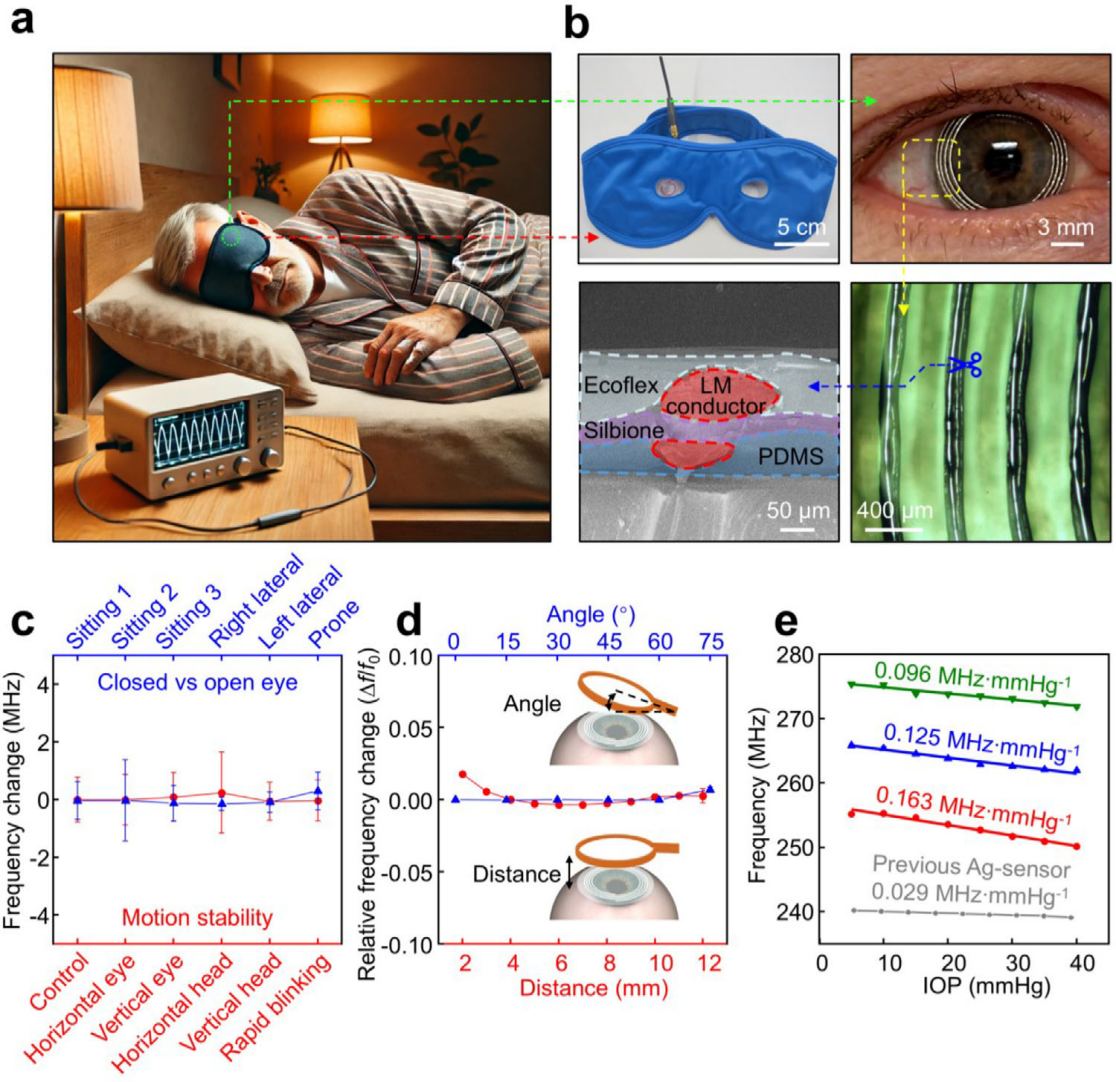
Closed‐eye, sleep‐like sensing capability. a, Overview of the sensor‐integrated soft contact lens and the sleep‐mask reader system designed for continuous IOP monitoring during sleep or rest. b, Photograph of the coil‐embedded sleep mask used for closed‐eye monitoring (top left), a subject wearing the sensor‐integrated contact lens (top right), an optical microscope image of the sensor (bottom right), and a cross‐sectional SEM image of the sensor (bottom left). c, Resonant‐frequency changes under various eye and head movements and during eye closure/opening. d, Relative frequency change in response to coil–sensor misalignment, across practical angular and distance variations. e, Ex vivo calibration results, compring to a previously reported Ag‐based design^22^.

Figure 1a illustrates the overall system concept for continuous nocturnal IOP monitoring. The schematic depicts a subject resting in a recumbent sleeping posture while wearing a soft contact lens sensor and a coil‐embedded sleep mask, representing a sleep‐relevant nocturnal monitoring configuration. In this configuration, corneal deformation modulates the resonance of the contact lens sensor, and the resulting pressure‐dependent signal is wirelessly coupled to a reader coil embedded in the sleep mask. The reader coil is connected to a bedside readout system for continuous signal recording, enabling overnight IOP monitoring across different eyelid states and moderate posture‐related variations. This setup supports both long‐term storage of nocturnal IOP profiles and real‐time monitoring, with the potential to trigger alerts when IOP exceeds clinically relevant thresholds during sleep.

Figure 1b (top panels) shows the key hardware components of the system, including a commercially available sleep mask with an integrated flexible receiver coil for stable wireless power and signal transfer under closed‐eye, recumbent conditions (Figure S1a), and a commercial soft contact lens embedding the wireless pressure sensor on the ocular surface (Figure S1b). The pressure sensor employs a multilayer LC resonant circuit for passive wireless monitoring of IOP‐induced corneal deformation, supporting comfortable closed‐eye operation during sleep without active user engagement [29]. Figure 1b (bottom panels) presents optical microscope and cross‐sectional scanning electron microscope (SEM) images illustrating the structure of the contact lens sensor. The LC resonant circuit is composed entirely of soft, elastomeric materials, including an LM (eutectic gallium–indium) conductor layer fully embedded between compliant dielectric and encapsulation layers (Figure S2). The circuit is designed as a thin annular structure (200 µm thick) with a narrow ring geometry (total width 1.5 mm, single‐turn width 150 µm) and is positioned at the lens periphery, outside the visual axis, to conform to the curved corneal surface while preserving the intrinsic properties of the commercial contact lens.

To evaluate robustness under realistic sleep‐like conditions, closed‐eye sensing performance was assessed in the presence of natural eye and head movements, as well as transitions between eye‐open and eye‐closed states. Across these conditions, resonant‐frequency fluctuations remained minimal, indicating negligible motion‐induced artifacts and stable signal acquisition during closed‐eye operation in healthy human eyes (Figure 1c), with raw data shown in Figure S3. Because practical overnight use also introduces variation in coil–sensor alignment, signal robustness was quantified across a range of angular offsets and separation distances representative of sleep‐mask wear [30]. Figure 1d shows that the relative frequency change (Δ*f*/*f*_0_) remained small across the tested conditions in an ex vivo pig eye model, demonstrating strong tolerance to coil misalignment and displacement. Figure 1e illustrates the ex vivo calibration results obtained in the pig eyes, yielding a sensitivity of 0.163 MHz mmHg^−1^ (*R* ^2^ = 0.977, or 639 ppm mmHg^−1^), representing a substantial improvement over the previously reported Ag‐based design [22]. In addition, an equivalent‐circuit‐based model is introduced to explain the enhanced sensitivity of the present device (Figure S4). The sensor is modeled as a pressure‐dependent RLC resonator inductively coupled to an external reader coil, where pressure‐induced deformation modulates the effective inductance and capacitance, thereby shifting the resonant frequency. Compared with the previously reported Ag‐based composite conductor, the liquid metal conductor better accommodates deformation while maintaining conductive continuity and minimizing dissipative loss, which may improve resonance quality and frequency‐shift transduction.

To further examine whether representative nearby RF sources could perturb the wireless readout, benchtop electromagnetic interference tests were performed by applying external RF signals adjacent to the sensor‐reader configuration. During frequency‐sweep testing from 50 to 800 MHz, the relative resonant frequency change remained negligible across the examined range (Figure S5a). Additional burst‐mode testing at 386, 433 MHz, 608–614 MHz, and 2 400 MHz, corresponding to the sensor‐relevant band, body sensor networks (BSN), wireless medical telemetry service (WMTS), and Wi‐Fi, respectively, likewise showed minimal perturbation of the measured signal during and after RF exposure (Figure S5b). These results suggest that the wireless readout is robust against representative nearby electromagnetic interference under the tested benchtop conditions. To further assess the compatibility of the sensor with ophthalmic medications relevant to the target patient population, benchtop drug‐exposure tests were performed using three anti‐glaucoma eye drops: Lumigan, Simbrinza, and Alphagan (Figure S6). The relative resonant frequency change remained minimal across all tested drug conditions, indicating that short‐term exposure to these representative topical glaucoma medications did not perturb the sensor readout under the tested benchtop conditions. The combination of high sensitivity and robust signal stability enables reliable detection of physiologically relevant IOP fluctuations under closed‐eye, sleep‐like conditions.

### 2.2 Quantitative Sensing Performance in an Ex Vivo Pig Eye Model

**FIGURE 2:**
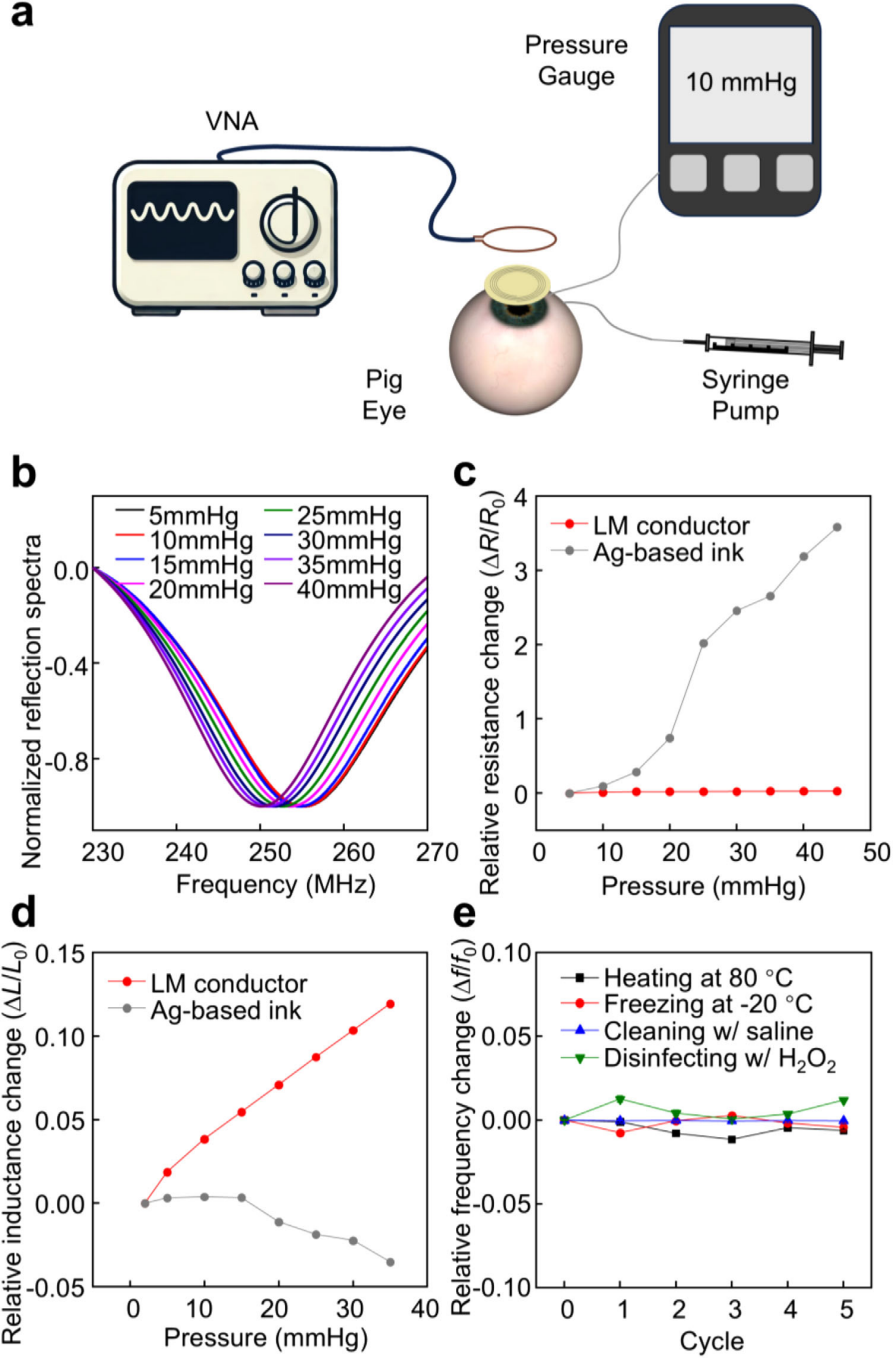
Quantitative sensing performance and engineering validation. a, Schematic illustration of the ex vivo experimental setup. b, Normalized reflection spectra demonstrating monotonic resonant‐frequency shifts in response to increasing IOP in the ex vivo eye model. c, Relative resistance change, and d, Relative inductance change for both traces under applied pressure mimicking physiologic IOP. e, Relative frequency change of the sensor following thermal cycling, cleaning, and disinfection, confirming environmental durability suitable for repeated clinical use.

To quantitatively evaluate sensor performance under controlled pressure conditions, we conducted electromechanical characterization using an ex vivo pig eye model that allows precise modulation of IOP [31]. Figure 2a illustrates the integrated experimental setup, in which IOP is adjusted via a syringe pump and pressure gauge while the contact lens sensor wirelessly couples to an external reader coil connected to a vector network analyzer (VNA). In the pig eye model, stepwise increases in IOP generated monotonic shifts in the normalized reflection spectra across the physiological range, confirming a stable pressure‐dependent response (Figure 2b). A representative image of this measurement setup is shown in Figure S7a. Linear fitting yielded high coefficients of determination, validating the suitability of this platform for quantitative calibration.

To evaluate the electromechanical advantages of the LM architecture, its performance was compared with that of an Ag‐based trace used in our earlier designs under both tensile and pressure loading. Under applied pressures spanning the physiological IOP range, the LM conductor exhibited substantially smaller resistance variation than the Ag‐based counterpart, indicating improved electrical stability under deformation (Figure 2c). Consistent with this behavior, the LM conductor showed a larger and more monotonic pressure‐dependent inductive response (Figure 2d). Tensile testing showed that the LM conductor maintained nearly constant resistance up to 50% strain (Figure S7b) and exhibited enhanced capacitive response under pressure (Figure S7c), indicating stronger electromechanical coupling and improved pressure sensitivity. The LM traces also preserved electrical integrity under repeated mechanical deformation, with stable resistance observed during cyclic stretching over thousands of cycles (Figure S7d). Deliberate overloading of the LM conductor embedded in PDMS revealed fracture at 0.84 MPa and 77% strain (Figure S7e), well above stresses expected during ocular wear or routine contact lens handling (∼1 kPa–350 kPa) [32]. In addition, daily baseline measurements during saline soaking storage showed only minimal relative resonant‐frequency drift over the tested period (Figure S8a). Cyclic loading/unloading tests under an applied compressive stress of approximately 1.5 kPa (∼10 mmHg), above the normal eyelid‐pressure range (∼8 mmHg) [32], further showed stable sensor response with negligible cycle‐dependent drift over 100 cycles (Figure S8b). Minimal resonant‐frequency drift was observed after thermal cycling, saline cleaning, and peroxide disinfection (Figure 2e). Additional benchtop testing showed minimal resonant‐frequency variation across temperatures from 20 to 50°C and humidity levels up to 90% RH (Figure S9), spanning a range broader than typical home‐use environments and supporting stable sensor readout under the tested conditions. Infrared (IR) thermal imaging showed negligible temperature increase after 12 h of continuous IOP monitoring on an enucleated pig eye (Figure S10). Together, these results demonstrate a substantial mechanical safety margin, durability under repeated clinical use, and negligible heat generation during prolonged overnight operation.

Because overnight IOP monitoring with a contact lens sensor inevitably involves lens displacement and deformation, the circuit geometry was optimized to balance electrical performance and mechanical compliance [33, 34]. Increasing the number of coil turns and reducing turn spacing shifted the resonance toward lower frequencies, improving coupling stability under closed‐eye conditions (Figure S11). In contrast, excessively dense geometries increased stiffness and hindered conformal transfer onto soft contact lenses [35]. Based on these trade‐offs, a four‐turn spiral with 450 µm spacing was selected for subsequent studies, providing robust resonance behavior while preserving lens softness. To assess the influence of lens material, sensors were integrated onto three commercially available soft contact lenses—AirOptix (Alcon, Inc.), Biofinity (CooperVision, Inc.), and Oasys (Johnson and Johnson Vision, Inc.) (Figure S12a). Marked differences in sensing performance were observed across lens types, suggesting that lens material properties, including stiffness, influence sensor transfer and signal fidelity [36]. Lenses with lower modulus are more susceptible to deformation during sensor transfer and handling [37]. Consistent with this trend, sensors integrated onto Oasys lenses, which have the lowest Young's modulus among the tested platforms, deformed during transfer and failed to maintain conformal attachment, resulting in degraded signal fidelity (Figure S12b). In contrast, AirOptix and Biofinity lenses, with higher modulus and rigidity, supported stable sensor integration and exhibited consistent pressure responses (Figure S12c). Key parameters and sensing performance across the three platforms are summarized in Table S2 [38, 39]. Given their stable sensing performance, favorable optometrist feedback, and prior use as the lens substrate in our previous work [22], AirOptix lenses were selected for subsequent in vivo studies.

### 2.3 Safety and Tolerability Under Prolonged Closed‐Eye Wear in Rabbits, Dogs, and Humans

**FIGURE 3:**
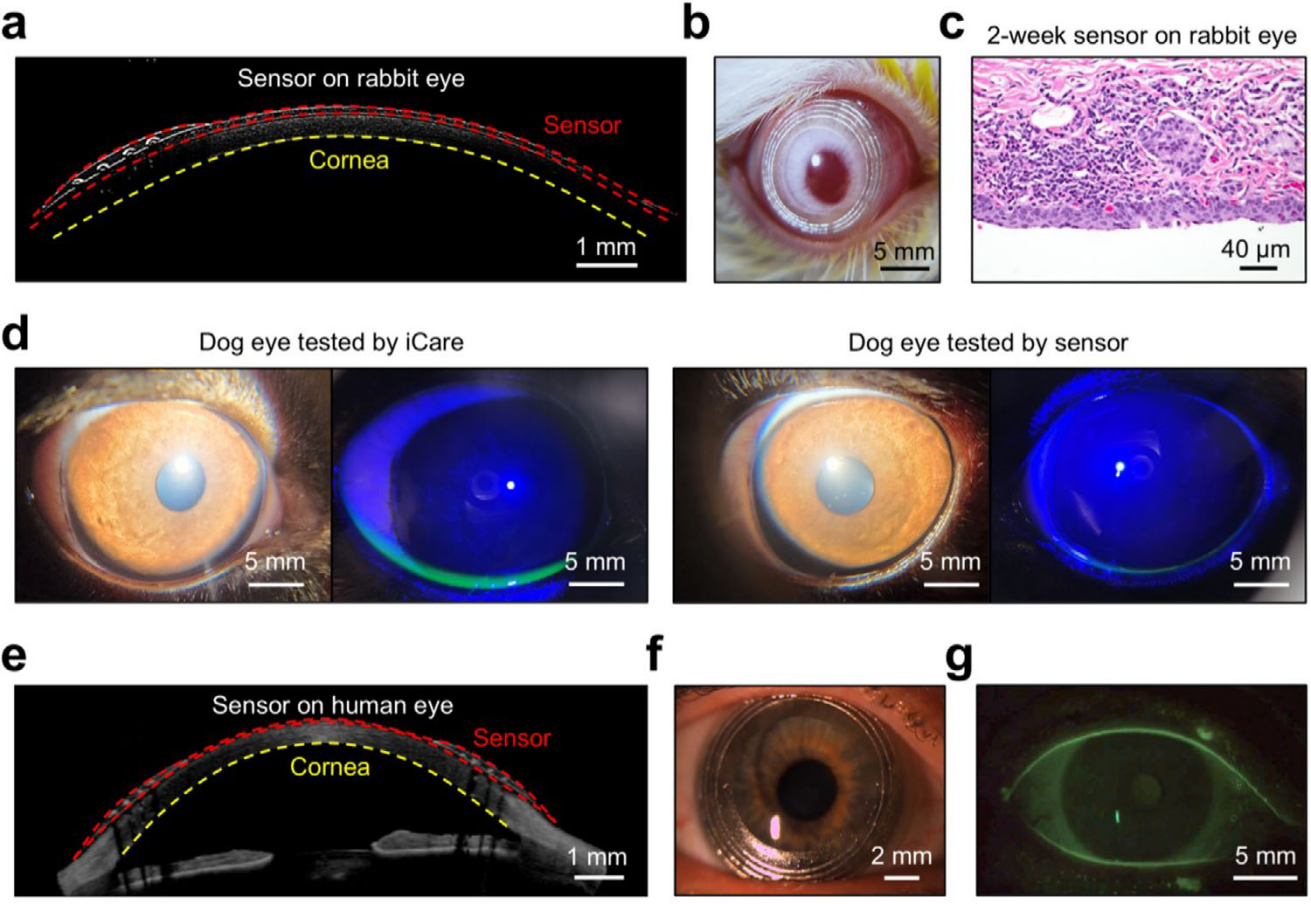
Safety and tolerability across rabbit, dog, and human subjects. a, AS‐OCT of a rabbit eye wearing the sensor, confirming conformal corneal attachment. b, Photograph of the rabbit eye with the sensor in place. c, Representative high‐magnification histological images from the 2‐week rabbit study. d, External ocular photographs with and without fluorescein staining of control (left) and sensor‐tested (right) dog eyes following sensor wear, confirming no corneal epithelial defects. e, AS‐OCT of the healthy subject wearing the sensor. f, Slit lamp photograph of a healthy human subject wearing the sensor. g, Post‐wear fluorescein staining in the healthy subject.

Because prolonged, closed‐eye wear is a prerequisite for sleep‐time IOP monitoring, the ocular safety and tolerability of the sensor were evaluated across multiple species [40, 41, 42]. In rabbits, the sensor conformed uniformly to the corneal surface, as confirmed by anterior segment optical coherence tomography (AS‐OCT) imaging (Figure 3a) and external photography (Figure 3b). In a 24‐h study, a contact lens with the sensor was applied to one eye and a bare lens to the other eye, and both were secured by partial eyelid closure using sutures to maintain positioning (Figure S13a). Post‐wear ocular surface imaging showed no visible abnormalities (Figure S13b), and inflammation scores revealed no notable differences between sensor‐wearing and control eyes (Table S3) [43, 44]. Histological analysis demonstrated only minimal to mild inflammation, characterized by scattered lymphocytes, plasma cells, and heterophils within the substantia propria and epithelium (Figure S14). The comparable findings between the sensor‐wearing and bare‐lens control eyes suggest that the observed mild ocular responses were associated not only with the integrated sensor, but also with lens wear itself, contact lens solution exposure, and procedure‐related handling factors [45, 46, 47, 48].

A two‐week study further evaluated tolerability under repeated daily wear, with the sensor applied for 8 h per day to one eye while the contralateral eye remained untreated. Figure 3c shows histopathological examination of the sensor‐wearing eye, revealing only minimal to mild inflammatory responses in the conjunctiva, comparable to those observed in the untreated contralateral control eye. Representative histopathological images from the sensor‐wearing and control eyes are shown in Figure S15a,b, respectively. To ensure stable sensor retention during the two‐week animal study, the third eyelid was surgically removed 1 week prior to sensor application (Figure S16a). Post‐study imaging and inflammation scores again showed no notable differences between eyes (Figure S16b).

We next evaluated the sensor in dogs, a clinically relevant large‐animal model for ophthalmic device testing [49]. Figure 3d shows slit‐lamp examination and fluorescein staining results demonstrating the absence of corneal epithelial defects following continuous sensor wear for up to 24 h, encompassing overnight, eyelid‐closed conditions. Sensor application followed standard soft contact lens procedures, enabling reproducible and atraumatic placement (Video S1). Further slit‐lamp evaluation revealed no evidence of intraocular inflammation (Figure S17a), while mild conjunctival hyperemia was observed in both eyes; it improved spontaneously within two days and fully resolved within one week (Figure S17b). In this model, the external reader coil was embedded in a custom‐fitted goggle to maintain stable coil–sensor alignment during measurements (Figure S18a). AS‐OCT imaging (Figure S18b) and external photography (Figure S18c) confirmed conformal attachment of the sensor to the corneal surface throughout the study period. Pharmacological reduction of IOP using topical latanoprost enabled calibration of the sensor‐derived signal (Video S2) against rebound tonometry in the contralateral eye (Video S3) [50]. This calibration yielded a strong linear relationship between resonant frequency and IOP with a sensitivity of 0.44 MHz mmHg^−1^, representing a substantial improvement over the previously reported Ag‐based platform (Figure S18d) [22]. Following calibration, the sensor was used for continuous IOP monitoring in dogs over a 24‐h period, with data acquired every 20 min. The resulting traces captured physiologically relevant diurnal IOP variations, including the overnight period from 6 p.m. to 6 a.m. (Figure S19a). Sensor‐derived IOP trends closely matched reference tonometry measurements, with strong agreement confirmed by limits‐of‐agreement analysis (Figure S19b).

In a healthy human subject, AS‐OCT imaging confirmed stable and conformal corneal contact during wear (Figure 3e), while slit‐lamp examination showed no evidence of epithelial disruption (Figure 3f). Figure 3g shows post‐wear fluorescein staining, which revealed intact corneal integrity without staining or infiltrates. A magnified slit‐lamp view in Figure S20 further illustrates partial tear‐film wetting over the sensor‐covered region and complete wetting elsewhere, without evidence of associated epithelial compromise [51]. The sensor could be self‐applied and removed using standard soft contact lens handling procedures (Video S4). These cross‐species results support the preliminary tolerability of the sensor under the tested short‐term and repeated closed‐eye wear conditions.

### 2.4 Usability and Comfort During Closed‐Eye Wear in Healthy and Glaucomatous Eyes

**FIGURE 4:**
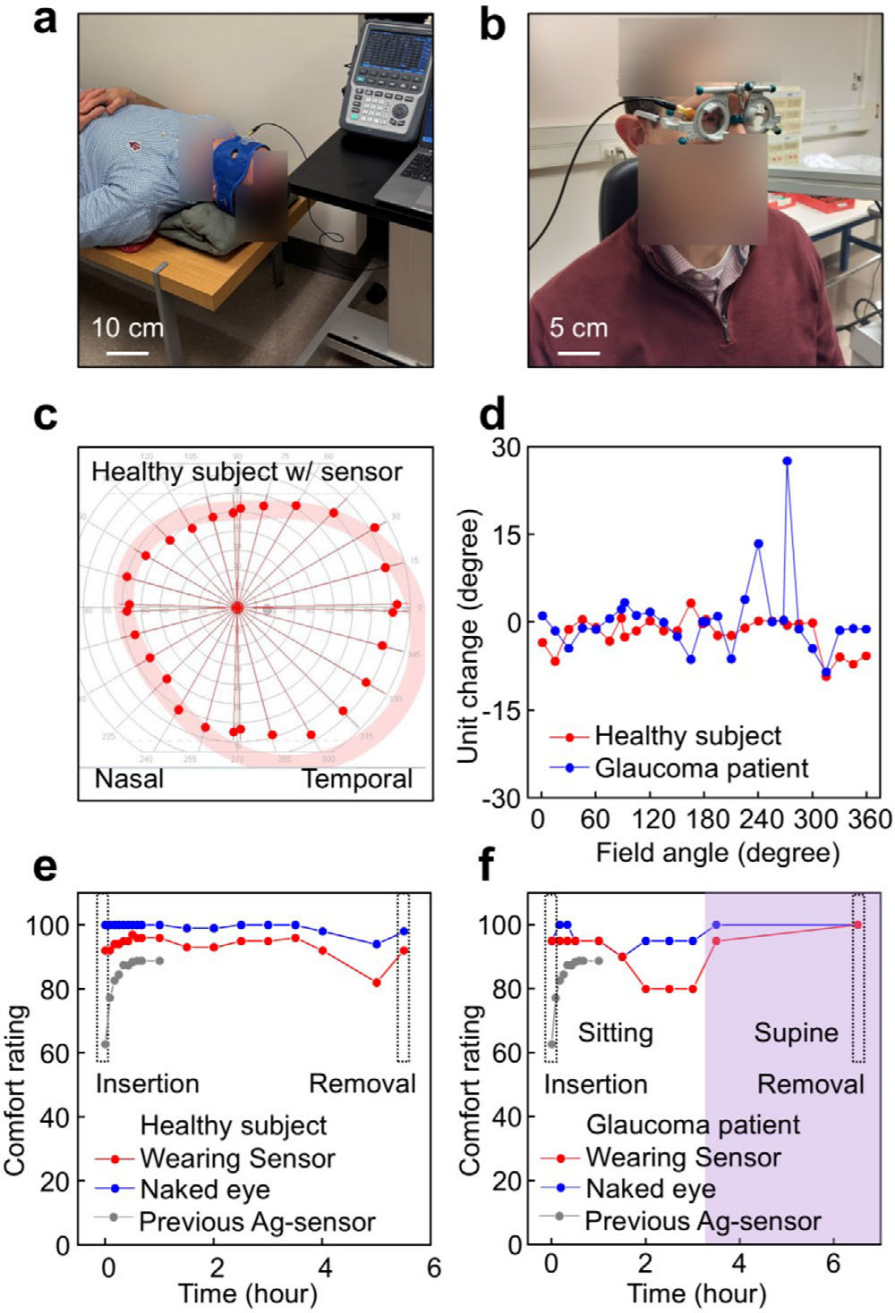
Usability and comfort in human subjects. a, Representative photograph of a subject wearing the coil‐embedded sleep mask in a supine, sleep‐mimicking posture. b, Representative image of a subject wearing the coil‐integrated trial frame during sitting, daily‐activity conditions. c, Visual‐field map of the sensor‐wearing eye in the healthy subject. d, Quantified visual‐field change for both the healthy subject and the glaucoma patient. e, Subjective comfort ratings of the healthy subject on a 0–100 scale, comparing to the previous Ag‐based design^22^. f, Subjective comfort ratings from the glaucoma subject, comparing to the previous Ag‐based design^22^.

To support closed‐eye IOP monitoring under realistic sleep conditions, the reader coil was primarily integrated into a wearable sleep mask designed for recumbent, closed‐eye (e.g., overnight) use (Figure 4a). For calibration and controlled testing, a coil‐integrated trial frame—a standard tool widely used in clinical optometry to precisely position lenses and optical components relative to the eye—was used as a temporary configuration to maintain a fixed coil–sensor distance, alignment, and viewing geometry under standardized conditions (Figure 4b) [52]. This setup enables reproducible measurements while minimizing variability arising from coil displacement or head motion, which is particularly important for establishing accurate calibration curves. Magnified photographs of both configurations are shown in Figure S21a, and the coil‐integrated trial frame is further illustrated in Figure S21b. Benchtop and human testing confirmed that both configurations exhibit equivalent resonance behavior, validating the trial frame as a calibration tool, while the sleep mask remains the primary interface for real‐world overnight monitoring (Figure S22a). The sensor also demonstrated stable signal characteristics under applied compressive loading that mimics eyelid closure, confirming reliable operation during closed‐eye wear (Figure S22b) [32].

Human usability and comfort were evaluated in both a healthy subject and a subject with pigmentary glaucoma. Figure 4c shows visual field testing in the healthy subject, demonstrating preservation of peripheral vision with the sensor in place, consistent with baseline measurements from the contralateral eye. Figure 4d illustrates a quantitative comparison of visual field measurements. Supporting assessments, including baseline data from the contralateral eye in the healthy subject (Figure S23a), as well as measurements from the naked eye (Figure S23b) and the sensor‐wearing eye in the glaucomatous subject (Figure S23c), revealed only mild reductions in the temporal and inferotemporal regions, all remaining within functional limits and without subjective visual distortion [53, 54]. These findings indicate preserved visual integrity during night‐time eye opening and upon awakening, supporting comfortable and compliant overnight monitoring.

Subjective comfort ratings, recorded on a 0–100 scale, remained high throughout monitoring in both a healthy subject and a subject with pigmentary glaucoma. The healthy subject reported scores generally above 90, with only a transient decrease during prolonged eye opening (Figure 4e) [55]. The glaucoma subject similarly reported sustained comfort, with ratings recovering to above 90 after transitioning to a recumbent, eyes‐closed posture (Figure 4f) [55]. In both cases, comfort scores compared favorably with those reported for earlier Ag‐based designs (Figure S24) [22], consistent with the fully encapsulated architecture and compliant soft‐conductor construction of the current sensor [56, 57]. To complement the subjective comfort scores, a simplified structural/mechanical analysis was also performed. Layer thicknesses estimated from the representative cross‐sectional SEM image, together with the corresponding material moduli, yielded an effective modulus of about 0.20 MPa for the multilayer sensor structure (Table S4), which was substantially lower than that of the AirOptix soft contact lens substrate (1.90 MPa). In addition, to provide an oxygen‐transmissibility‐related assessment of mass transport across the sensor‐integrated lens, we performed a benchtop water vapor transmission assay (Figure S25). After 144 h, the cumulative water vapor transmission of the sensor‐integrated lens was 0.077 ± 0.003 g cm^−2^, corresponding to 77% of that of the bare lens and remaining higher than that of the fully Kapton‐covered barrier control. Together, these results support the feasibility of comfortable, closed‐eye wear during sleep, a critical requirement for continuous nocturnal IOP monitoring in clinical practice.

### 2.5 Clinical and Posture‐Dependent IOP Dynamics in Glaucoma Subjects

**FIGURE 5:**
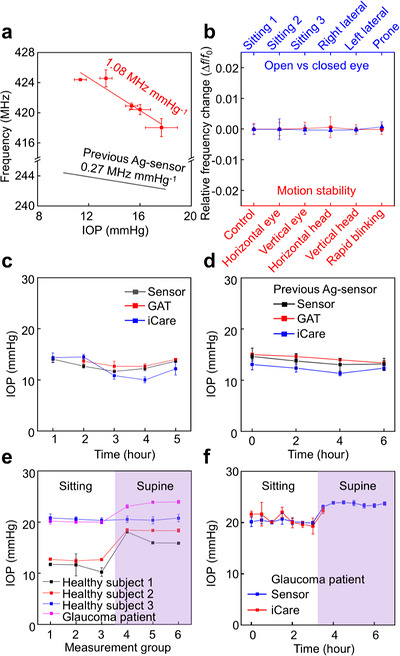
Clinical physiology and posture‐dependent IOP dynamic. a, Calibration curve generated by correlating sensor‐derived resonant frequency with iCare measurements with comparison to the prior Ag‐based sensor^22^. b, Relative resonant frequency changes under various eye and head movements and with the eye open or closed across multiple postures. c, Diurnal IOP measurements in the healthy subject obtained using the sensor, iCare, and GAT. d, Reference diurnal IOP measurement data reproduced from a prior Ag‐based platform for comparison^22^. e, Summary of posture‐dependent IOP changes in all healthy subjects and the glaucoma patient before and after the sitting‐to‐supine transition. f, Posture‐dependent IOP measurements in the glaucoma patient, consistent with physiologically relevant nocturnal IOP increases.

To establish a reference framework for interpreting posture‐dependent sensor responses under nocturnal monitoring conditions, posture‐dependent calibration was first performed in a healthy subject by correlating sensor‐derived resonant frequency with iCare‐measured IOP in the contralateral eye across multiple body postures. This pre‐established relationship was subsequently used to estimate posture‐related IOP changes in additional human measurements, as described below. All figures and movies involving human subjects were reproduced using a healthy volunteer in accordance with IRB privacy requirements, even when representing procedures or measurements performed in glaucoma patients. Figure 5a shows the resulting calibration curve, which exhibited a linear relationship, resonant frequency (MHz) = 437.57 – 1.08 × IOP (mmHg), corresponding to a sensitivity of 1.08 MHz mmHg^−1^ (*R* ^2^ = 0.868), or 2 542 ppm mmHg^−1^. Representative normalized reflection spectra associated with this calibration are shown in Figure S26a. The sensor‐derived resonant‐frequency measurements are illustrated in Video S5, while the corresponding contralateral‐eye tonometry procedure across postures is shown in Video S6. This sensitivity represents a substantial improvement over our earlier designs and exceeds values reported for other soft contact lens sensors [22]. The sensitivity values varied across ex vivo pig‐eye, in vivo dog‐eye, and human measurements, likely reflecting differences in ocular biomechanics, coupling conditions, and measurement configuration that affect strain‐transfer efficiency and the resulting frequency response [58]. Based on this clinical calibration sensitivity, the practical resolution was further estimated from stable lens‐mounted bench‐top traces to be approximately 0.83 mmHg (3σ; Table S5).

Sensor robustness was next evaluated under conditions emulating overnight wear, including ocular and head motion, as well as transitions between eye‐open and eye‐closed states across multiple postures (Figure 5b). Across all conditions, relative frequency variations remained below 0.1%, with standard deviations below 0.5%, comparable to baseline fluctuations and substantially smaller than the multi‐megahertz shifts associated with physiological IOP changes. Representative footage of the measurements across different motion conditions is provided in Video S7. Pressure‐dependent signals remained robust under eyelid contact, recumbency, and incidental motion, supporting reliable closed‐eye IOP monitoring during sleep. To assess temporal tracking performance, diurnal IOP fluctuations were monitored in the healthy subject over a 5‐h daytime period as a reference for physiological pressure dynamics. Sensor‐derived IOP trends closely matched both Goldmann applanation tonometry (GAT) and iCare measurements (Figure 5c), demonstrating agreement comparable to that of our prior soft contact lens sensors while offering improved sensitivity and suitability for sleep‐relevant measurements (Figure 5d) [22]. Posture‐dependent IOP changes were further evaluated using a sitting‐to‐supine transition protocol in the original healthy subject, two additional healthy subjects, and a subject with glaucoma. Sensor‐derived IOP was estimated using the pre‐established calibration relationship described above. Recumbency‐associated IOP elevation is a recognized contributor to nocturnal IOP burden yet remains difficult to capture clinically [59, 60]. In the original healthy subject, healthy subject 2, and the glaucoma subject, sensor‐derived IOP increased following transition from sitting to supine posture, consistent with the corresponding tonometry‐based trend (Figure 5e). In contrast, healthy subject 3 showed minimal posture‐dependent IOP change. This low postural responsiveness is consistent with reported inter‐subject variability in recumbency‐induced IOP changes, influenced by episcleral venous pressure, aqueous outflow, ocular rigidity, and choroidal vascular compliance [61, 62], and was consistently observed by both the sensor and iCare measurements. These findings support the ability of the sensing approach to capture subject‐dependent posture‐related IOP behavior across independent human measurements. Representative normalized reflection spectra are shown for the glaucoma subject (Figure S26b) and the original healthy subject (Figure S26c), confirming robust signal transduction under sleep‐mimicking conditions. Figure S26d summarizes time‐resolved posture‐dependent IOP data from the healthy subject, showing good agreement with GAT measurements in the sitting posture. Additional time‐resolved measurements from the two added healthy subjects are provided in Figures S27 and S28 further summarizes supporting clinical assessments, including slit‐lamp images of the sensor on eye (Figure S28a), AS‐OCT confirmation of sensor position and corneal separation (Figure S28b), comfort ratings during and after wear (Figure S28c), and quantified visual‐field changes across field angles (Figure S28d). Representative footage of sensor application and removal is provided in Video S8.

In the glaucoma subject, extended monitoring was performed under both sitting and supine conditions to assess IOP dynamics relevant to nocturnal elevation. Figure 5f shows a distinct and sustained increase in sensor‐derived IOP following transition from sitting to supine posture, consistent with physiologically relevant nocturnal IOP elevation observed in glaucoma [63]. During the sitting phase, reference IOP measurements were obtained from the contralateral eye using iCare; during the supine phase, reference measurements were limited to the onset of recumbency to better emulate natural sleep conditions, when tonometry is not routinely feasible. Throughout the monitoring period, the participant engaged in typical daily activities, including reading, eating, and smartphone use (Figure S29). Representative footage of these activities is provided in Video S9. AS‐OCT and slit‐lamp imaging confirmed stable sensor positioning throughout these activities (Figure S30). Minor conjunctival hyperemia observed on slit‐lamp examination was attributed to pre‐visit glaucoma medication rather than sensor wear (Figure S31). These results highlight the platform's ability to capture pressure dynamics inaccessible in routine clinical practice.

## 3 Discussion

In this work, we introduce a soft contact lens sensor with a sleep‐mask reader that enables continuous, high‐fidelity monitoring of nocturnal IOP. Enhanced sensitivity and an optimized device architecture enable robust IOP measurement under recumbent, closed‐eye conditions while accommodating normal ocular and head motion. Conformal integration onto commercial soft contact lenses preserves lens curvature, wearer comfort, and corneal biomechanics, supporting real‐world feasibility for overnight use. Together, these features allow subtle nocturnal IOP fluctuations to be resolved, with motion‐ and eye‐closure–related variations remaining substantially smaller than IOP‐driven signals.

Safety evaluations in rabbits, dogs, and human subjects demonstrated stable corneal contact, preserved epithelial integrity, and good wearer comfort, supporting suitability for prolonged closed‐eye use. Building on this safety foundation, clinical physiology measurements underscore the translational relevance of the platform. In a glaucoma subject, the sensor enabled reliable IOP monitoring during extended wear and revealed a sustained elevation in IOP under supine, eye‐closed conditions that mimic nocturnal sleep—a physiologically important yet clinically inaccessible window in current practice. Real‐time quantification of posture‐ and sleep‐related IOP dynamics provides new insight into nocturnal IOP burden and may inform individualized risk assessment and therapeutic decision‐making. The agreement between sensor‐derived and tonometry‐based trends across subjects supports the feasibility of capturing subject‐dependent IOP behavior under the tested conditions.

From a translational perspective, this platform builds on commercially available soft contact lenses, a reader coil compatible with standard sleep masks, and scalable fabrication processes based on dispenser printing that support batch production for clinical deployment and eventual at‐home use. The present system should be viewed as a prototype research platform rather than a fully optimized untethered home‐use device. Although stable sensing is demonstrated under tested recumbent, closed‐eye, and motion‐related conditions, fully unconstrained overnight motion during natural sleep remains to be evaluated. Further miniaturization and reduced tethering will improve wearability and facilitate broader home deployment. With further development of portable readout electronics, the system could support autonomous data acquisition and wireless transmission, enabling continuous IOP monitoring throughout daily life—using a lightweight eyewear frame during waking hours and a sleep mask configuration overnight. Expanded clinical studies across diverse patient populations, including different glaucoma subtypes, together with refined calibration strategies that account for interindividual variability, will further strengthen clinical translation. Overall, this work establishes a clinically adaptable soft contact lens sensor for practical assessment of nocturnal IOP dynamics beyond the reach of conventional glaucoma management.

## 4 Methods

### 4.1 Sensor Fabrication

A glass slide (Premium Plain Microscope Slides, Fisher Scientific, Inc.) was spin‐coated with a 10 wt.% polyvinyl alcohol (PVA; Mowiol 4–88) solution at 2 000 rpm for 30 s and annealed at 80 °C for 2 h. Printable inks were prepared as follows: polydimethylsiloxane (PDMS) ink by mixing base components (Dowsil SE 1700 and Sylgard 184; Dow Corning, Inc.) and a curing agent at a weight ratio of 10:10:1; LM conductor ink using eutectic gallium–indium (EGaIn, Sigma‐Aldrich, Inc.); Silbione ink by mixing components A and B of Silbione RT Gel 4717 (Bluestar Silicones); Ecoflex ink was prepared by mixing components A and B of Ecoflex 00–31 Near Clear (Smooth‐On, Inc.) with Silicone Thinner (Smooth‐On, Inc.) at a weight ratio of 10:10:1.6. All ink formulations were homogenized using a planetary centrifugal mixer (ARE‐310, Thinky, Inc.).

Layer‐by‐layer printing was performed using an automated nozzle dispensing system (Nordson EFD) mounted on a three‐axis translation stage with ≥100 µm resolution and ±3 µm repeatability. Nozzle speeds ranged from 5–25 mm s^−1^, depending on ink viscosity. All layers were cured at 80 °C for 2 h, while the LM conductor layers remained uncured. To prevent leakage due to the fluidity of EGaIn, the entire patterned structure was encapsulated with Ecoflex. The printed sensor was then released from the substrate by dissolving the PVA layer in deionized water.

A thin polydopamine (PDA) adhesive layer was formed by floating the released sensor overnight on Tris buffer (10 mM, pH 8.5) containing dopamine hydrochloride (2 mg mL^−1^; Sigma‐Aldrich, Inc.), followed by transfer onto commercial soft contact lenses and annealing at 40°C for 2 h [64].

Prior to each experiment, sensors were disinfected for at least 8 h using a commercial disinfecting solution (Biotrue Multi‐Purpose Solution, Bausch & Lomb, Inc.) per the manufacturer's instructions. Several commercially available soft contact lenses were used in this study, including Air Optix Night & Day Aqua (Alcon, Inc.), Acuvue Oasys (Johnson & Johnson, Inc.), and Biofinity (CooperVision, Inc.).

### 4.2 Benchtop Evaluation

Optical images of the sensor were captured using an optical microscope (Eclipse LV150N, Nikon), and cross‐sectional SEM images were captured using a high‐resolution SEM (S‐4800, Hitachi). IR thermal images were captured using a high‐resolution infrared camera (A300, FLIR).

Standard tensile tests were performed using a mechanical tester (ESM303, Mark‐10), while electrical resistance was measured using a source meter (Keithley 2400, Keysight, Inc.). For Ag‐based controls, Ag/TPU composite ink was prepared by dissolving 1.48 g TPU (Elastollan C60AW) in 3.76 g tetrahydrofuran (THF, Sigma‐Aldrich, Inc.) and 4 g dimethylformamide (DMF, Sigma‐Aldrich, Inc.), then blending with 5.96 g Ag flakes (2–5 µm, Inframat Advanced Materials, Inc.) using a centrifugal mixer [65]. Sensors were applied to enucleated pig eyes. IOP was controlled via saline injection using a syringe pump (Micro4, World Precision Instruments, Inc.), and pressure was measured with a gauge (HHP 452, Omega, Inc.). Resistance, inductance, and capacitance were measured using a precision LCR meter (E4980AL, Keysight, Inc.). Relative changes in resistance, inductance, and capacitance (Δ*R*/*R*_0_, Δ*L*/*L*_0_, Δ*C*/*C*_0_) were calculated as the difference between current value and initial value over the initial value, with initial value defined at the minimum applied pressure (e.g., 5 mmHg).

For misalignment studies, sensors were mounted on an enucleated pig eye with the reader coil fixed above. Relative frequency change (Δ*f*/*f*_0_) was calculated as the difference between current frequency and initial frequency over the initial frequency, with initial frequency defined at 0° angle or 4 mm distance.

Environmental robustness was evaluated by: heating at 80 °C for 30 min followed by resting at room temperature for 1 h; freezing at −20 °C for 30 min followed by resting at room temperature for 1 h; rinsing with saline (Renu, Bausch & Lomb, Inc.); and disinfecting with hydrogen peroxide solution (Clear Care, Alcon, Inc.) for five cycles, respectively.

### 4.3 Electromagnetic Interference Test

The susceptibility of the wireless sensing system to external RF interference was evaluated using an RF signal generator (MAX2870) positioned adjacent to the sensor‐reader setup. For frequency‐sweep testing, continuous RF signals were applied from 50 to 800 MHz with 1 MHz increments and a dwell time of 3 s at each frequency. To further assess interference at representative frequencies, short RF burst tests were performed at 386, 433 MHz, 608–614 MHz, and 2,400 MHz, corresponding to the sensor‐relevant band, body sensor networks (BSN), wireless medical telemetry service (WMTS), and Wi‐Fi, respectively. During burst‐mode testing, RF exposure was applied from 6 to 14 s while the sensor signal was continuously recorded for 20 s with a 2 s interval. Relative frequency change was calculated as (*f_t_* − *f*_0_)/*f*_0_, where *f*_0_is the initial resonant frequency measured before application of external RF interference and *f_t_* is the resonant frequency measured at each time point or test condition.

### 4.4 Ophthalmic Drug Exposure Test

To evaluate the effect of representative topical anti‐glaucoma medications on sensor performance, benchtop drug‐exposure tests were conducted using sensors transferred onto soft contact lenses. The lens‐mounted sensors were first placed in a commercial lens case and immersed in saline to establish the baseline resonant frequency. Three commercially available glaucoma eye drops were then sequentially tested: Lumigan (0.01% bimatoprost ophthalmic solution; AbbVie, Inc.), Simbrinza (1%/0.2% brinzolamide/brimonidine tartrate ophthalmic suspension; Alcon, Inc.), and Alphagan (0.1% brimonidine tartrate ophthalmic solution; Allergan, Inc.). For each condition, the drug solution was applied to cover the lens surface, and the sensor signal was recorded. The lens was then rinsed with saline and remeasured before proceeding to the next drug condition. For each data point, three measurements were taken and averaged. Relative frequency change was calculated with respect to the saline baseline.

### 4.5 Ambient Environmental Stability Test Under Varying Temperature and Humidity

To evaluate the effect of ambient temperature and humidity on sensor readout, additional benchtop environmental stability tests were performed. For temperature testing, the resonant frequency of the sensor‐reader system was first recorded at room temperature (20°C) as the baseline, and then remeasured after the system was exposed to ambient temperatures of 30, 40, and 50°C. Relative frequency change was calculated with respect to the 20°C baseline.

For humidity testing, the laboratory ambient condition (approximately 40% RH) was used as the baseline. Ambient humidity was then increased stepwise up to 90% RH using a commercial cool mist humidifier (DREO, Inc.), and the corresponding humidity values were recorded from the humidifier display. The resonant frequency was measured at each humidity condition, and relative frequency change was calculated with respect to the baseline condition.

### 4.6 Ex Vivo Evaluation

Enucleated pig eyes were placed in Petri dishes. Two 23G needles (BD PrecisionGlide, BD, Inc.) were inserted into the anterior chamber: one connected to the syringe pump for IOP control, and the other to a pressure gauge for real‐time monitoring. The sensor was conformally applied to the cornea. The reader coil was positioned above the eye and connected to a VNA (N9913A FieldFox, Keysight, Inc.).

Reflection spectra (S11) were continuously recorded and normalized to a range of ‐1 to 0. For each data point, three measurements were taken and averaged. Sensitivity in MHz mmHg^−1^ or ppm mmHg^−1^ was calculated as:

(1) $$\mathrm{Sensitivity}\;\left(\mathrm{MHz}\;\mathrm{mmH}g^{-1}\right)={\left|\frac{\partial f}{\partial P}\right|}_{P=P_{\min}}\;$$

(2) $$\mathrm{Sensitivity}\;\left(\mathrm{ppm}\;\mathrm{mmH}g^{-1}\right)={\left|\frac{\partial f}{\partial P}\right|}_{P=P_{\min}}/f\left(P=P_{\min}\right)$$

Where *f* is the resonant frequency, *P* is the IOP value, *P*_min_ is the minimum IOP value in the experiment, and *f* (*P* = *P*_min_) represents the resonant frequency at the minimum IOP value.

### 4.7 Long‐Term Baseline Stability, Repetitive Loading Stability, and Practical‐resolution Characterization

For long‐term baseline‐stability testing, the sensor was stored in saline solution, and the baseline signal was recorded daily during storage. Relative resonant‐frequency change was calculated with respect to the day 0 measurement. For repetitive loading stability characterization, cyclic loading/unloading tests were performed by repeatedly applying a compressive stress of approximately 1.5 kPa (∼10 mmHg) to the sensor, which is higher than the normal eyelid‐pressure range (∼8 mmHg). The sensor signal was recorded after loading and after unloading in each cycle, and a total of 100 cycles were performed.

For practical‐resolution estimation, the sensor was positioned under fixed readout geometry relative to the reader coil and continuously measured without applied pressure variation. Resonant frequency was extracted from each scan. For each trace, 150 data points were used for analysis. The practical resolution was estimated as:

(3) $$\mathrm{Practical}\;\mathrm{resolution}\;\left(\mathrm{mmHg}\right)=3\sigma_{f}/|S|$$

Where *σ*_*f*_is the standard deviation of the baseline resonant‐frequency fluctuation and *S*is the calibration sensitivity. Three stable lens‐mounted bench‐top traces were analyzed in this manner.

### 4.8 Water Vapor Transmission Assay

A benchtop water vapor transmission assay was performed as an oxygen‐transmissibility‐related assessment of mass transport across the sensor‐integrated lens. 5 mL glass vials were filled with 3 mL DI water and covered with one of the following conditions: a bare AirOptix soft contact lens, a sensor‐integrated AirOptix soft contact lens, no cover as an uncovered control, or Kapton tape as a barrier control. The total weight of each vial assembly was measured every 24 h for up to 144 h. Cumulative water vapor transmission was calculated by dividing the weight loss by the effective evaporation area of the vial opening. Data are presented as mean ± s.d. from three independent samples for each condition (*n* = 3).

### 4.9 In Vivo Evaluation in Rabbit Eyes

All procedures complied with the Association for Research in Vision and Ophthalmology (ARVO) Statement for the Use of Animals in Ophthalmic and Vision Research and were approved by the Purdue Animal Care and Use Committee (Protocol #2104002129). Six 6‐month‐old New Zealand white rabbits (Envigo Global Services, Inc.) were included and housed under a 12‐h light/dark cycle.

Three rabbits were used for a 24‐h biocompatibility study, with the sensor fitted on one eye and a bare lens on the contralateral eye. Both eyelids were partially sutured (4‐0 Ethilon Nylon, Ethicon, Inc.) to maintain lenses in place. Fluorescein staining (Ful‐Glo, Akorn, Inc.) and slit‐lamp biomicroscopy (SL‐17, Kowa, Inc.) were performed before and after wear. AS‐OCT imaging (Spectralis, Heidelberg Engineering, Inc.) was used to confirm sensor alignment.

The remaining three rabbits were used for a 2‐week study, with the sensor fitted on one eye and the contralateral eye served as untreated control. Third eyelids were removed one week in advance to allow lens retention. After confirming ocular health, sensors were worn 8 h per day for 2 weeks, with daily disinfection using Biotrue (Bausch and Lomb, Inc.). Eyes were monitored daily for irritation or inflammation. Rabbits and eye assignments were randomized.

At study endpoints, rabbits were humanely euthanized, and ocular tissues were collected, fixed, paraffin‐embedded, and stained with H&E. Histopathology was assessed using a masked, semi‐quantitative scoring scale (0/0.5/1/2/3: no lesions, minimal, mild, moderate, and severe).

### 4.10 In Vivo Evaluation in Dog Eyes

All procedures adhered to ARVO guidelines and were approved by the Purdue Animal Care and Use Committee (Protocol #2104002129). Sensor calibration was performed using iCare Tonovet every 2 h, applied to the contralateral eye to minimize perturbation. Latanoprost (0.005%, Bausch & Lomb, Inc.) was used to induce IOP variation. Four measurements per time point were averaged for empirical linear fitting.

On a separate day, 24‐h continuous IOP monitoring was conducted using both the sensor and iCare Tonovet at 20‐min intervals. The animals were not sacrificed after the measurement.

### 4.11 Human Subject Studies

Human studies were conducted with Institutional Review Board (IRB) approval (Protocol #24139) in accordance with Good Clinical Practice (GCP) and institutional ethical guidelines. All participants provided written informed consent and were provided ($40) hourly monetary compensation for their participation. Due to IRB regulations, all images and movies associated with patient data could not be used directly and were instead reenacted by co‐author researchers for illustration purposes.

OCT imaging was performed in corneal high‐resolution mode across 16 meridians. Sensor positioning and ocular surface health were evaluated by slit‐lamp biomicroscopy (SL120, Zeiss, Inc.). Visual field extent was quantified with an Octopus 900 Kinetic Perimeter (Haag‐Streit, Inc.) with a III4e target (64 mm^2^ area, 318 cd m^−2^ luminance, 5° s^−1^ target speed). The shaded pink region marks the 25^th^–75^th^ percentile range for age‐matched normal individuals. The unit change in visual field was calculated as the difference between the values recorded with the sensor in place and those obtained under baseline (naked‐eye) conditions.

Sensor calibration was performed using an iCare Home tonometer (iCare USA, Inc.) under various body postures. For all IOP measurements obtained via GAT and iCare Home, the contralateral eye was used to avoid direct corneal perturbation of the sensor‐wearing eye. Data were collected after a 5‐min rest period to allow IOP stabilization, and each data point represented the mean of four consecutive measurements.

For the motion effect study, the subject remained seated throughout the experiment. Baseline control data were first recorded with the subject stationary, followed by sequential measurements during horizontal eye movement, vertical eye movement, horizontal head movement, vertical head movement, and rapid blinking. The resonant frequency change was calculated as the difference between the frequency recorded during each motion condition and the control baseline. Four measurements were averaged per data point.

For eye opening and closure study, measurements were obtained with the eye open and then with the eye closed at each posture. Postures included sitting, right lateral, left lateral, and prone orientations. The resonant frequency change was determined as the difference between the open‐eye and closed‐eye states. Three groups of data were collected at sitting posture, and four measurements were averaged per data point.

For continuous IOP monitoring under a single posture, the subject remained seated for a 5‐h measurement session, with IOP readings obtained using the sensor, iCare Home, and GAT, with 1‐h intervals. For continuous IOP monitoring across wakeful and sleep‐mimicking conditions in the glaucoma patient, the subject first maintained a sitting posture and then transitioned to a supine posture. Resonant frequency data were acquired at seven time points for each posture at 30‐min intervals, yielding a total of 14 measurements. A similar posture‐transition protocol was applied to an additional healthy subject, but with shortened sampling intervals of 15 min in the sitting posture and 5 min in the supine posture. Four measurements were averaged per data point.

## Author Contributions

S.A.P., P.S.K., and C.H.L. conceived the concept; planned the project; and supervised the research. Y.D., Y.L., F.L., J.L., and C.H.L. designed, fabricated, and characterized the sensor. Y.O. provided mechanistic analysis of the sensor. Y.D., T.M.L., F.L., K.L., D.M.S., P.S.K., and C.H.L. designed and conducted clinical studies. Y.D., O.T.O., F.L., Z.W., T.P., H.R., S.A.P., and C.H.L. designed and conducted in vivo evaluations in rabbits and dogs. Y.D., T.Y., and S.H. conducted SEM characterization. G.C.S conducted histological analysis of the rabbit eyes. Y.D., F.L., and C.H.L. wrote the manuscript. All authors commented on the manuscript.

## Conflicts of Interest

The authors declare no conflicts of interest.

## Trial Registration

This clinical study was registered at ClinicalTrials.gov under identifier NCT07224542.

## Supporting information




**Supporting File 1**: adma73813‐sup‐0001‐SuppMat.docx.

**Supporting File 2**: adma73813‐sup‐0002‐MovieS1.mp4.

**Supporting File 3**: adma73813‐sup‐0003‐MovieS2.mp4.

**Supporting File 4**: adma73813‐sup‐0004‐MovieS3.mp4.

**Supporting File 5**: adma73813‐sup‐0005‐MovieS4.mp4.

**Supporting File 6**: adma73813‐sup‐0006‐MovieS5.mp4.

**Supporting File 7**: adma73813‐sup‐0007‐MovieS6.mp4.

**Supporting File 8**: adma73813‐sup‐0008‐MovieS7.mp4.

**Supporting File 9**: adma73813‐sup‐0009‐MovieS8.mp4.

**Supporting File 10**: adma73813‐sup‐0009‐MovieS9.mp4.

## Data Availability

All data are available in the main text or the supplementary materials.
