# Supplementary material for: Nocturnal Intraocular Pressure Monitoring With a Soft Contact Lens Sensor for Glaucoma Management
Source: Adv Mater. 2026 Jun 23;38(42):e73813. doi: 10.1002/adma.73813 (PMC13410647; doi:10.1002/adma.73813)
Supplement: Supplementary file 1 — Supporting File 1: adma73813‐sup‐0001‐SuppMat.docx. [file ADMA-38-e73813-s002.docx]

Supplementary Materials for

**Nocturnal intraocular pressure monitoring with a soft contact lens sensor for glaucoma management**

Yumin Dai^†^, Tristan Michael Long^†^, Oluwabunmi T. Oladele^†^, Youngoh Lee^†^, Feiyang Li, Ziheng Wang, Yeonji Oh, Junsang Lee, Taewoong Park, Tianhao Yu, Seokkyoon Hong, Kyeonghee Lim, Jinheon Jeong, Dawn Meyer Schneider, Hyerim Ra, Bryan W. Boudouris, Gillian C. Shaw, Shin Ae Park^*^, Pete S. Kollbaum^*^, Chi Hwan Lee^*^

Corresponding author: Shin Ae Park, park1222@purdue.edu; Pete S. Kollbaum, kollbaum@iu.edu; Chi Hwan Lee, lee2270@purdue.edu

**The PDF file includes:**

Supplementary Figs. 1-31

Supplementary Tables 1-5

**Other Supplementary Materials for this manuscript include the following:**

Supplementary Videos 1-9


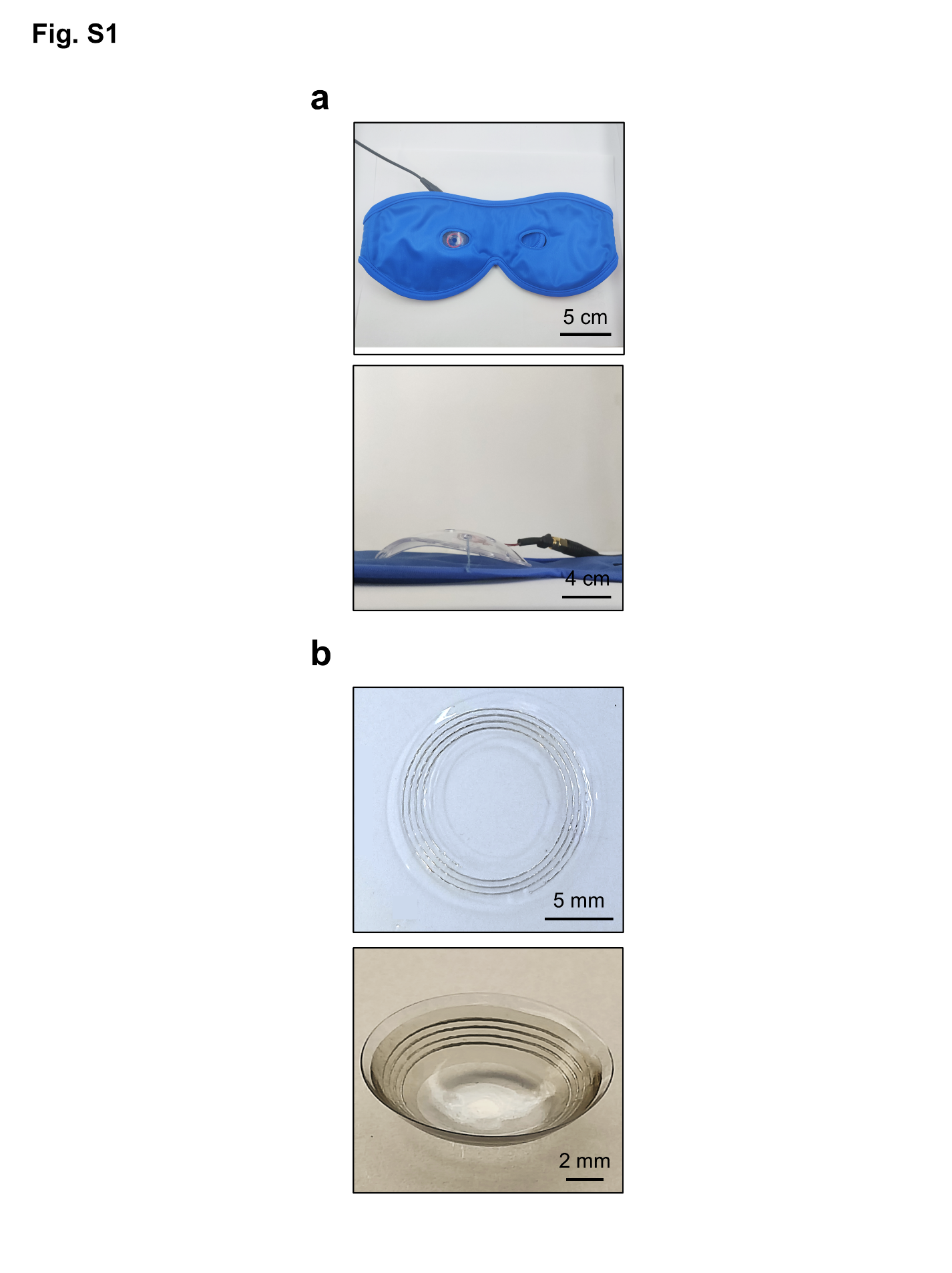


Supplementary Fig. 1: Additional images of sensor and sleep mask. (a) Front view (top) and side view (bottom) of the coil-embedded sleep mask. (b) Photograph of printed sensor (top) and the sensor transferred onto commercial soft contact lens (bottom).


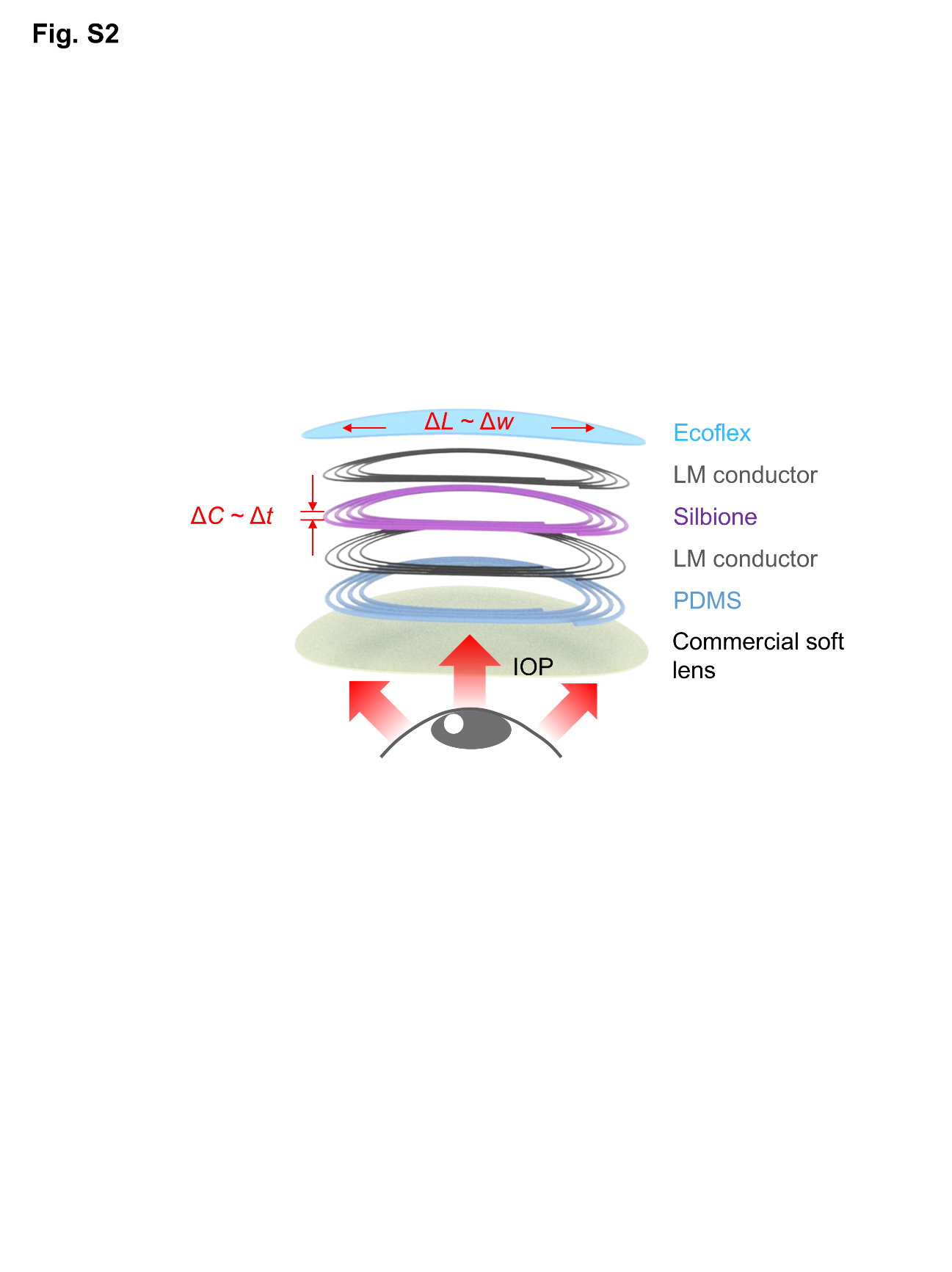


Supplementary Fig. 2: Schematic of the layered structure of the sensor, demonstrating the LC resonant sensing mechanism.


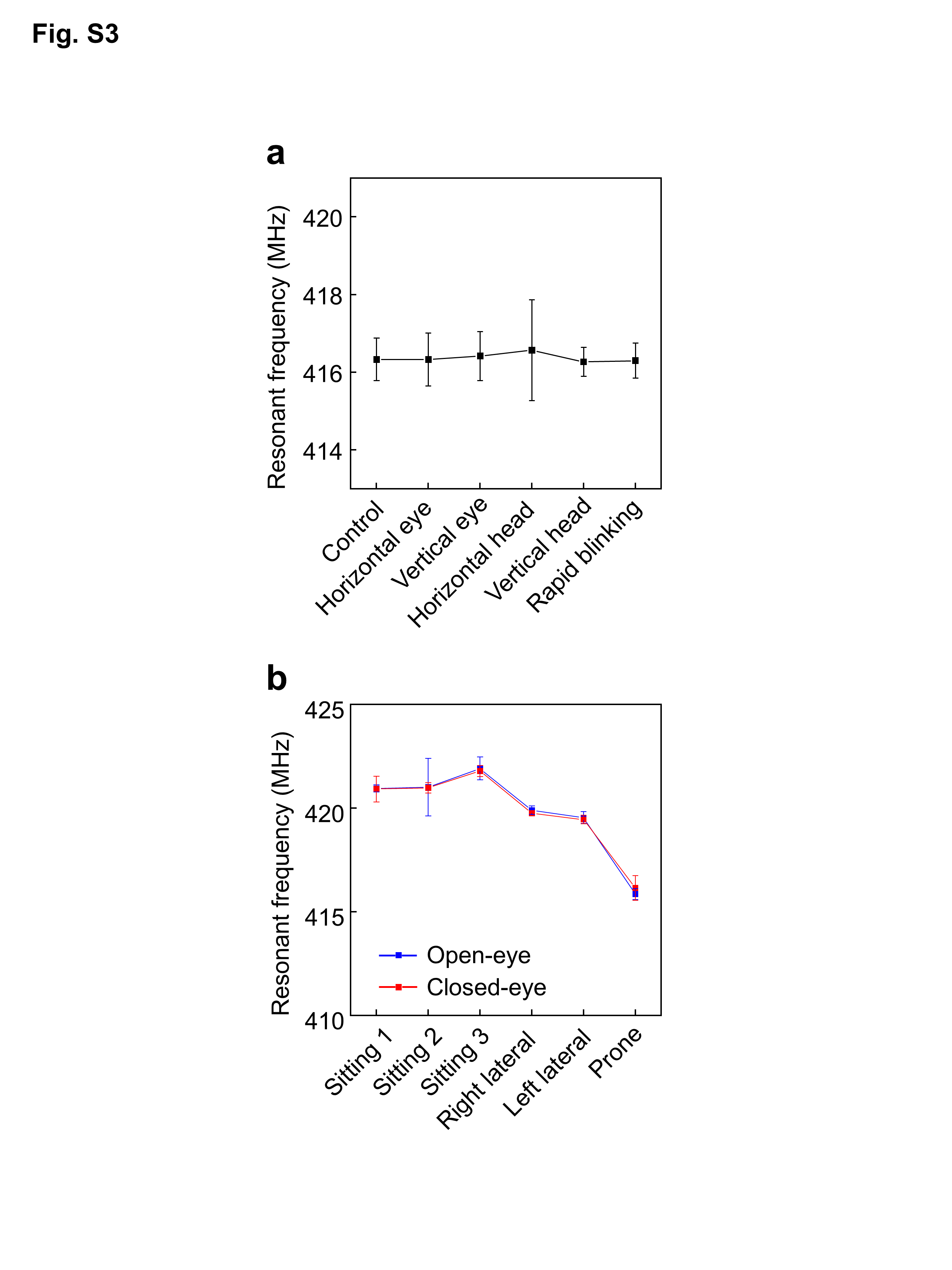


Supplementary Fig. 3: Additional data of motion effect and open/close-eye effect study. (a) Resonant frequency under different motion conditions. (b) Resonant frequency under different postures with eye open and closed.


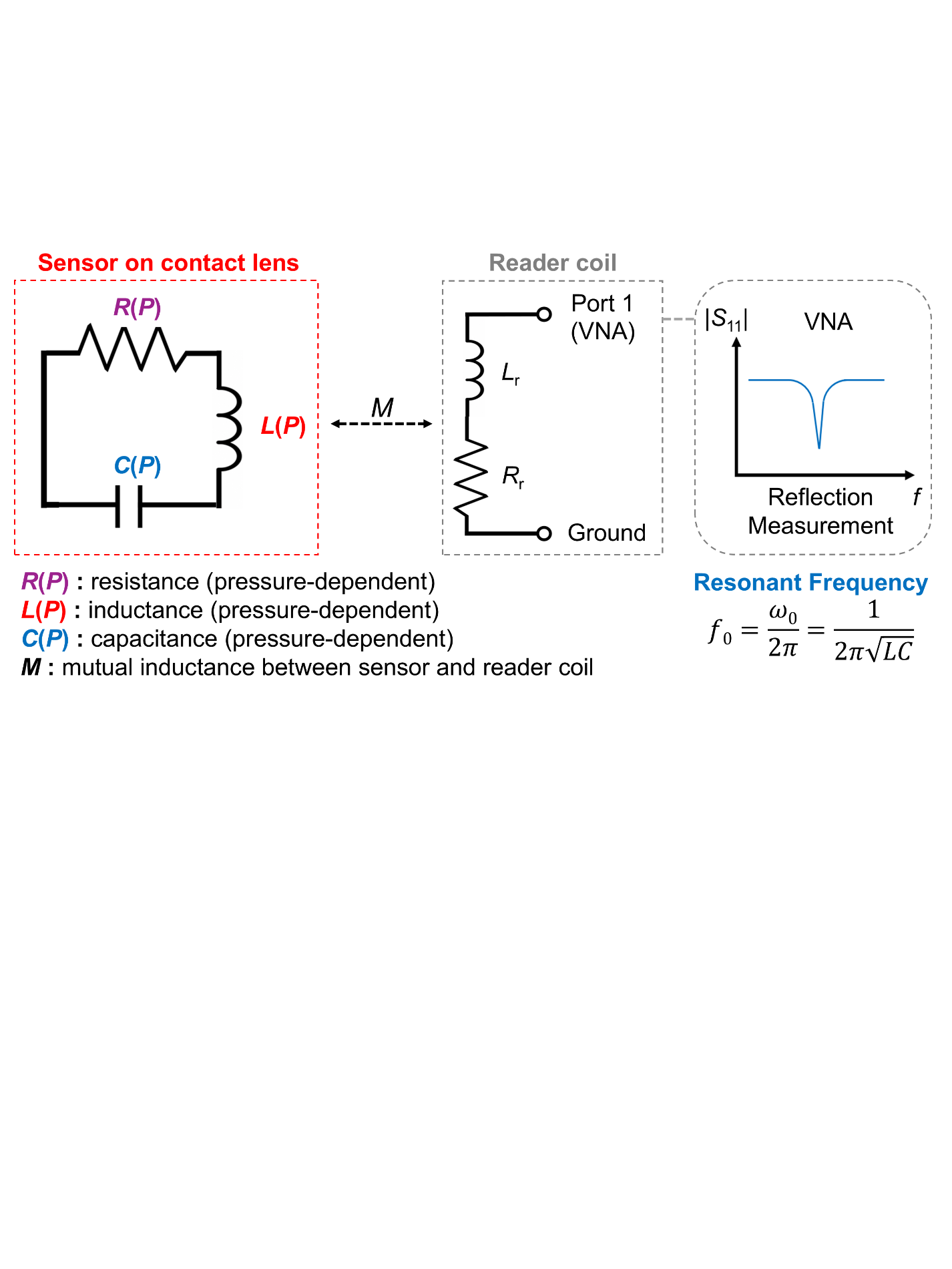


Supplementary Fig. 4: Equivalent-circuit-based mechanistic model of the liquid-metal soft contact lens sensor. The sensor integrated on the contact lens is modeled as a pressure-dependent RLC resonator with resistance $\boldsymbol{R(P)}$, inductance $\boldsymbol{L(P)}$, and capacitance $\boldsymbol{C(P)}$, inductively coupled to the external reader coil through mutual inductance $\boldsymbol{M}$. Pressure-induced deformation modulates the effective resonant elements of the sensor, resulting in a shift in resonant frequency, which is detected from the reflected $\boldsymbol{S}_{\boldsymbol{11}}$signal of the vector network analyzer.


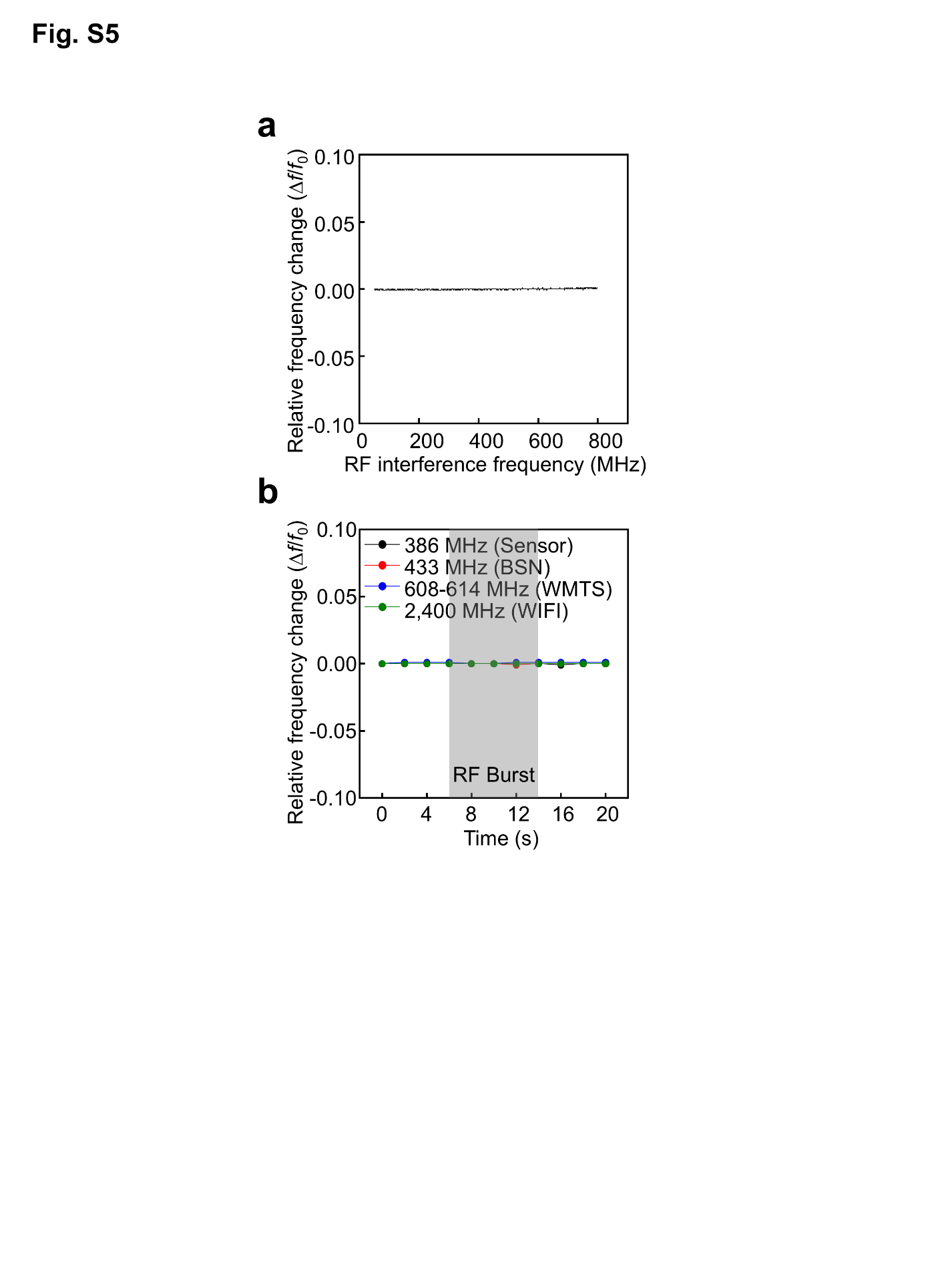


Supplementary Fig. 5: Electromagnetic interference test of the wireless contact lens sensor under representative external RF exposure. (a) Relative resonant-frequency change during frequency-sweep RF interference testing from 50 to 800 MHz, showing negligible variation across the tested range. (b) Time-course of relative resonant-frequency change during short RF burst exposure at representative frequencies relevant to the sensor operating band, BSN, WMTS, and Wi-Fi. The shaded region indicates the RF burst period. Minimal signal perturbation was observed under all tested conditions.


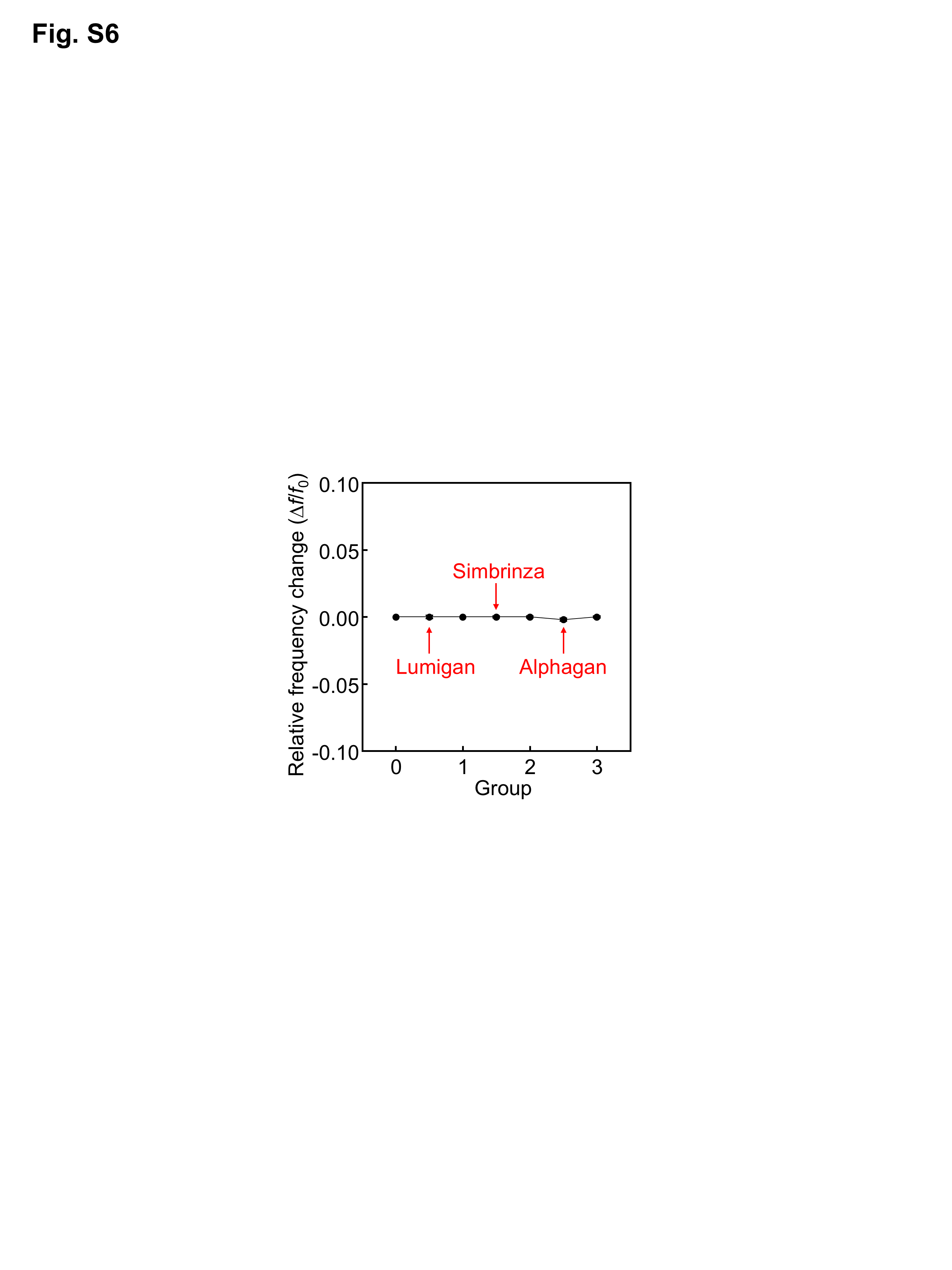


Supplementary Fig. 6: Benchtop evaluation of sensor response under exposure to representative topical anti-glaucoma medications. Relative resonant frequency change of the sensor after sequential exposure to three glaucoma eye drops: Lumigan (0.01% bimatoprost ophthalmic solution), Simbrinza (1%/0.2% brinzolamide/brimonidine tartrate ophthalmic suspension), and Alphagan (0.1% brimonidine tartrate ophthalmic solution). The sensor was rinsed with saline and remeasured after each drug application.


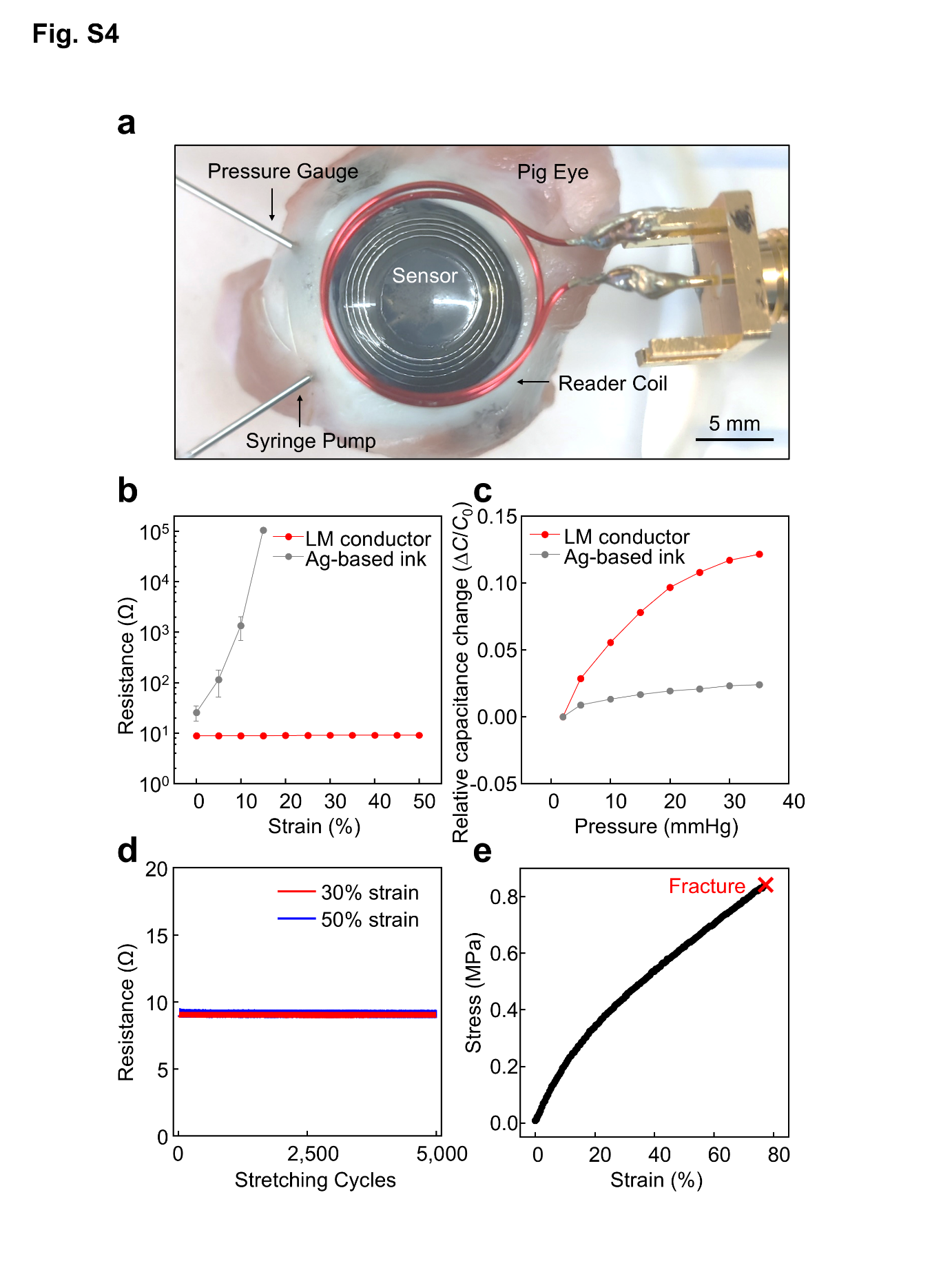


Supplementary Fig. 7: Ex vivo and benchtop evaluation results. (a) Photograph of the ex vivo evaluation on an enucleated pig eye. (b) Resistance of LM conductor and Ag-based traces under tensile strain. (c) Relative capacitance change for both traces under applied pressure mimicking physiologic IOP. (d) Electrical stability of the soft-conductor trace during repeated stretching cycles. (e) Stress-strain curve of the LM conductor embedded in PDMS.


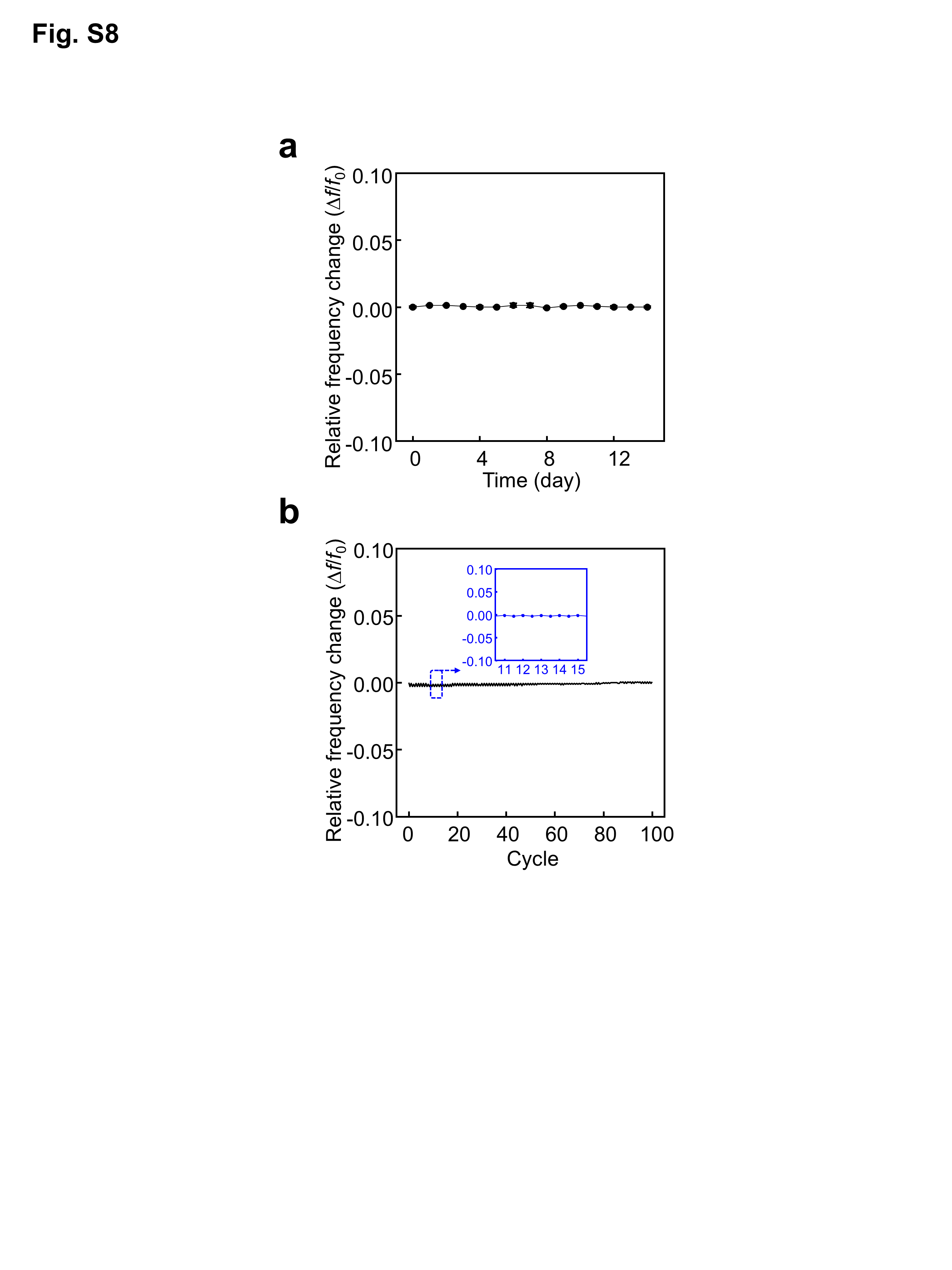


Supplementary Fig. 8: Benchtop evaluation of long-term baseline stability and repetitive loading stability. (a) Relative resonant-frequency change of the sensor during saline soaking storage, measured daily and normalized to the day 0 value. (b) Relative resonant-frequency change during cyclic loading/unloading under a compressive stress of ~1.5 kPa (~10 mmHg), exceeding the normal eyelid-pressure range (~8 mmHg). The signal was recorded after loading and unloading in each cycle over 100 cycles. Inset shows cycles 11–15.


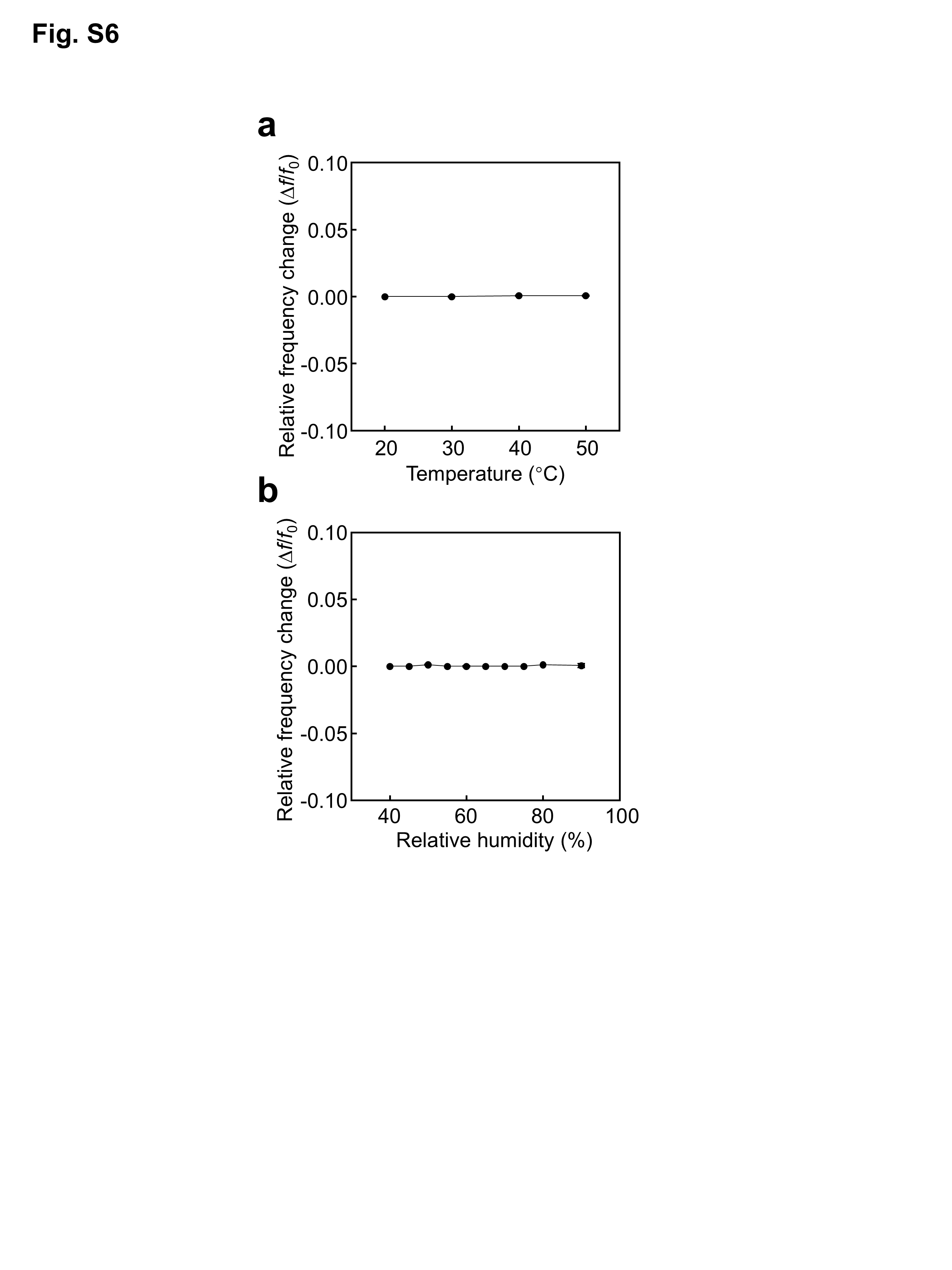


Supplementary Fig. 9: Benchtop evaluation of sensor frequency stability under varying ambient temperature and humidity conditions. (a) Relative resonant frequency change measured at elevated ambient temperatures of 30, 40, and 50 °C, using 20 °C as the baseline condition. (b) Relative resonant-frequency change measured under ambient humidity up to 90% RH, using the laboratory default condition (~40% RH) as the baseline. Humidity was adjusted using a commercial cool mist humidifier, and values were recorded from the device display.


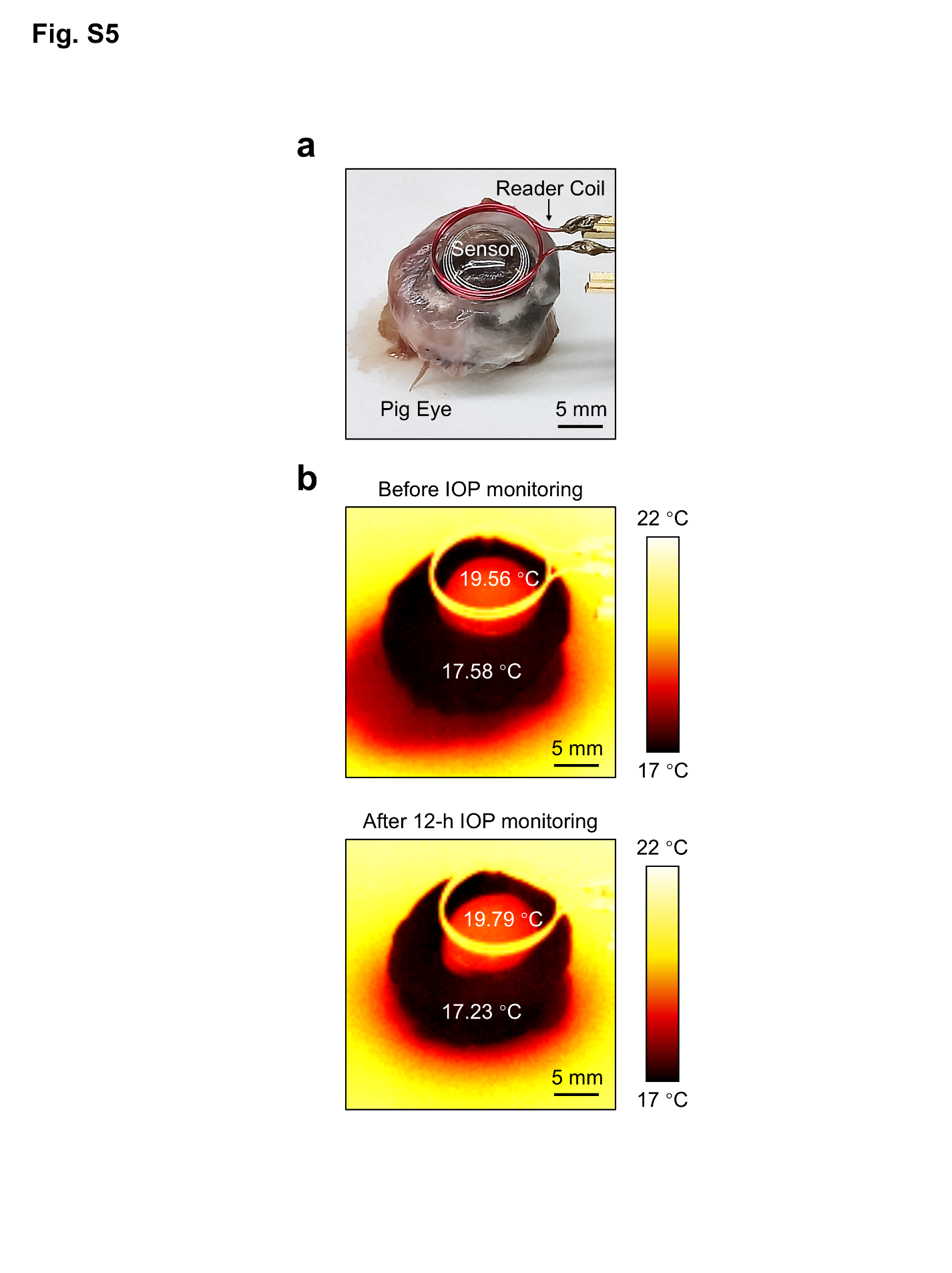


Supplementary Fig. 10: Infrared thermal evaluation of the sensor during prolonged operation. (a) Photograph of the sensor mounted on an enucleated pig eye with the reader coil for measurement. (b) Infrared thermal images before and after 12 h of IOP monitoring, showing representative temperatures at the sensor and surrounding tissue.


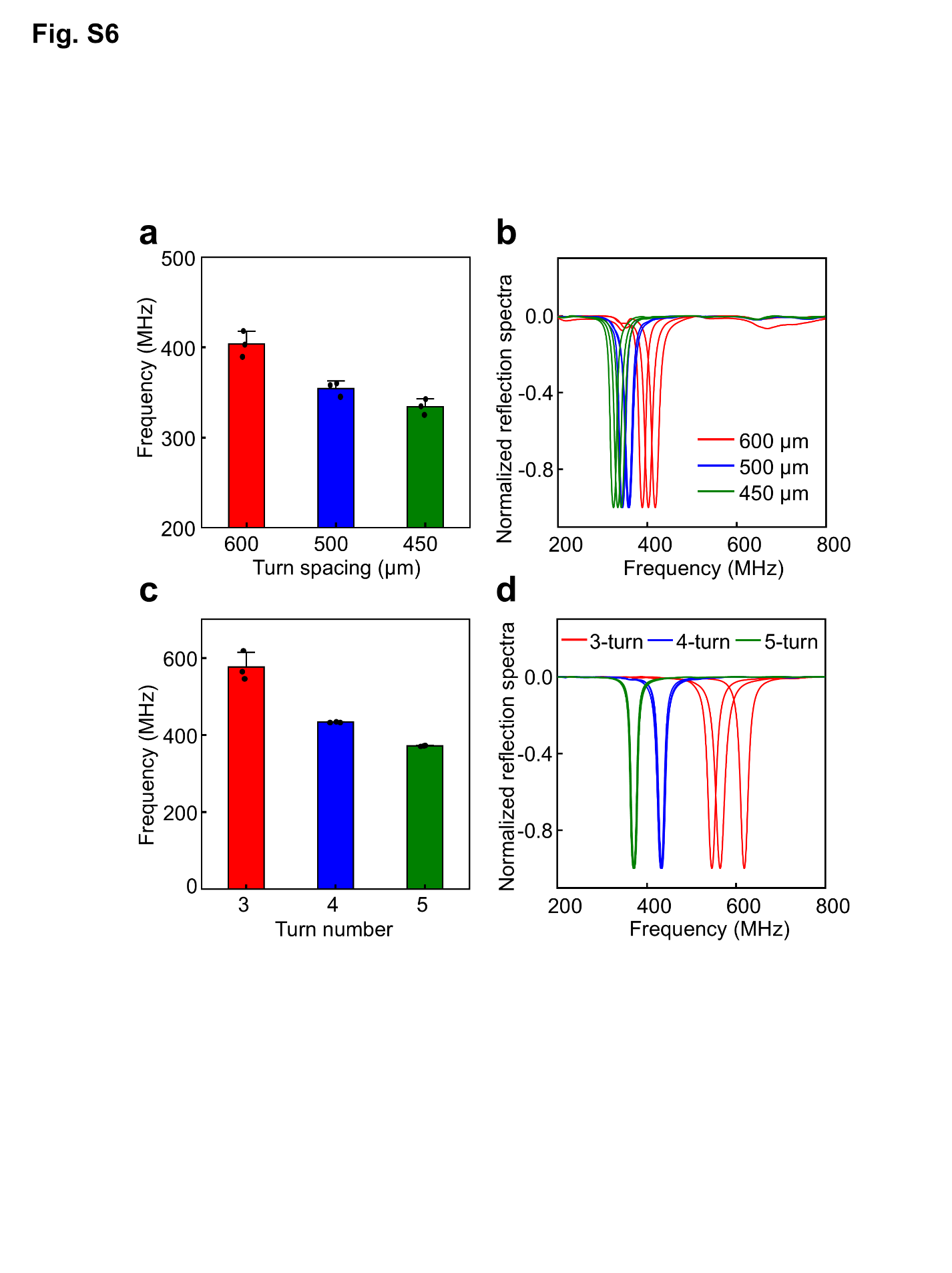


**Supplementary Fig. 11: Turn spacing and turn number effect in sensor design.** (**a**) Frequency in response to different turn spacing, and (**b**) corresponding normalized reflection spectra. (**c**) Frequency in response to different turn number, and (**d**) corresponding normalized reflection spectra.


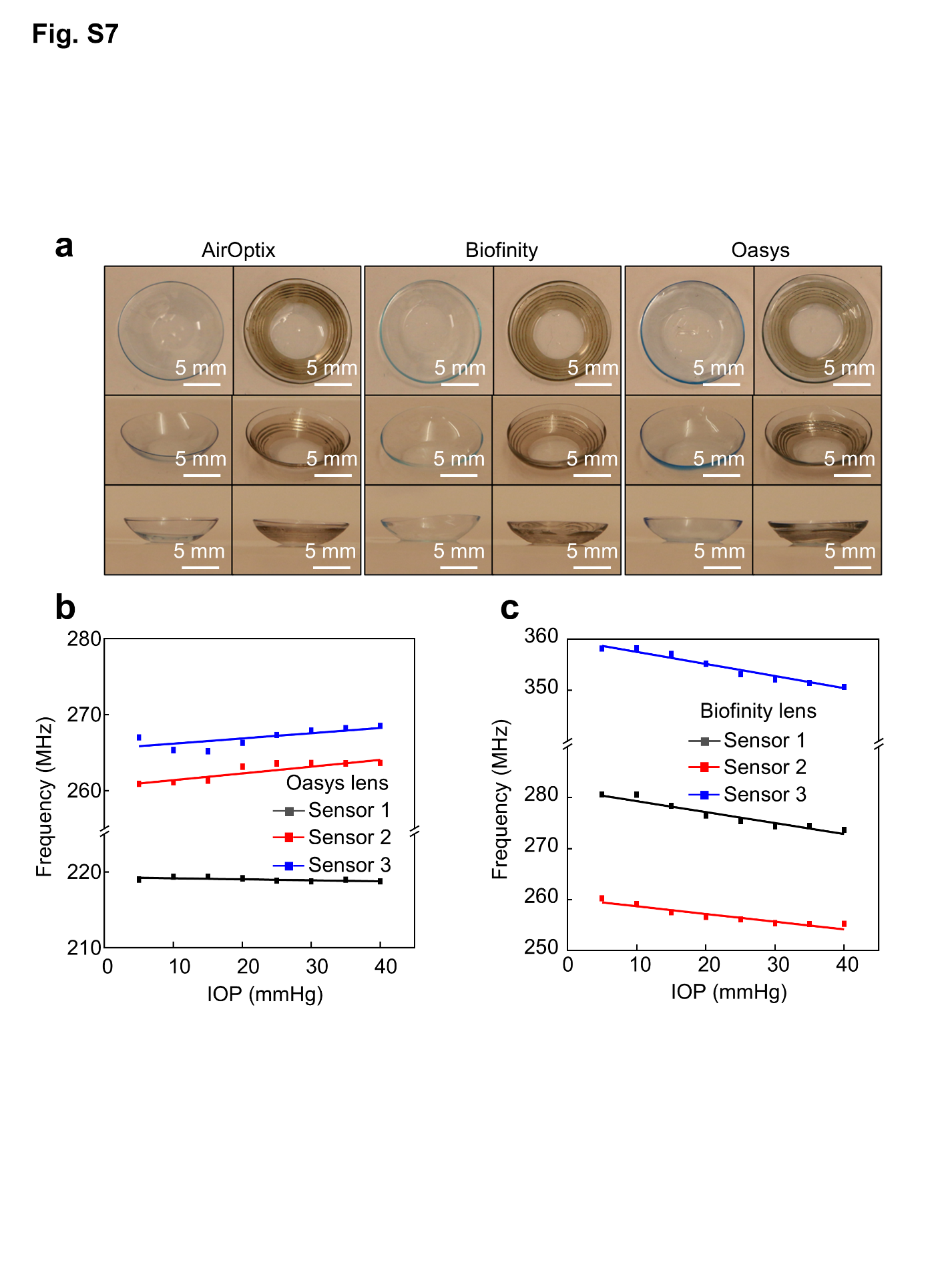


Supplementary Fig. 12: Evaluation of sensors on different brands of soft contact lenses. (a) Images of sensor on different brands of contact lenses compared with bare lenses: AirOptix (left), Biofinity (mid), and Oasys (right). (b) Calibration curve of sensors on Oasys lenses. (c) Calibration curve of sensors on Biofinity lenses.


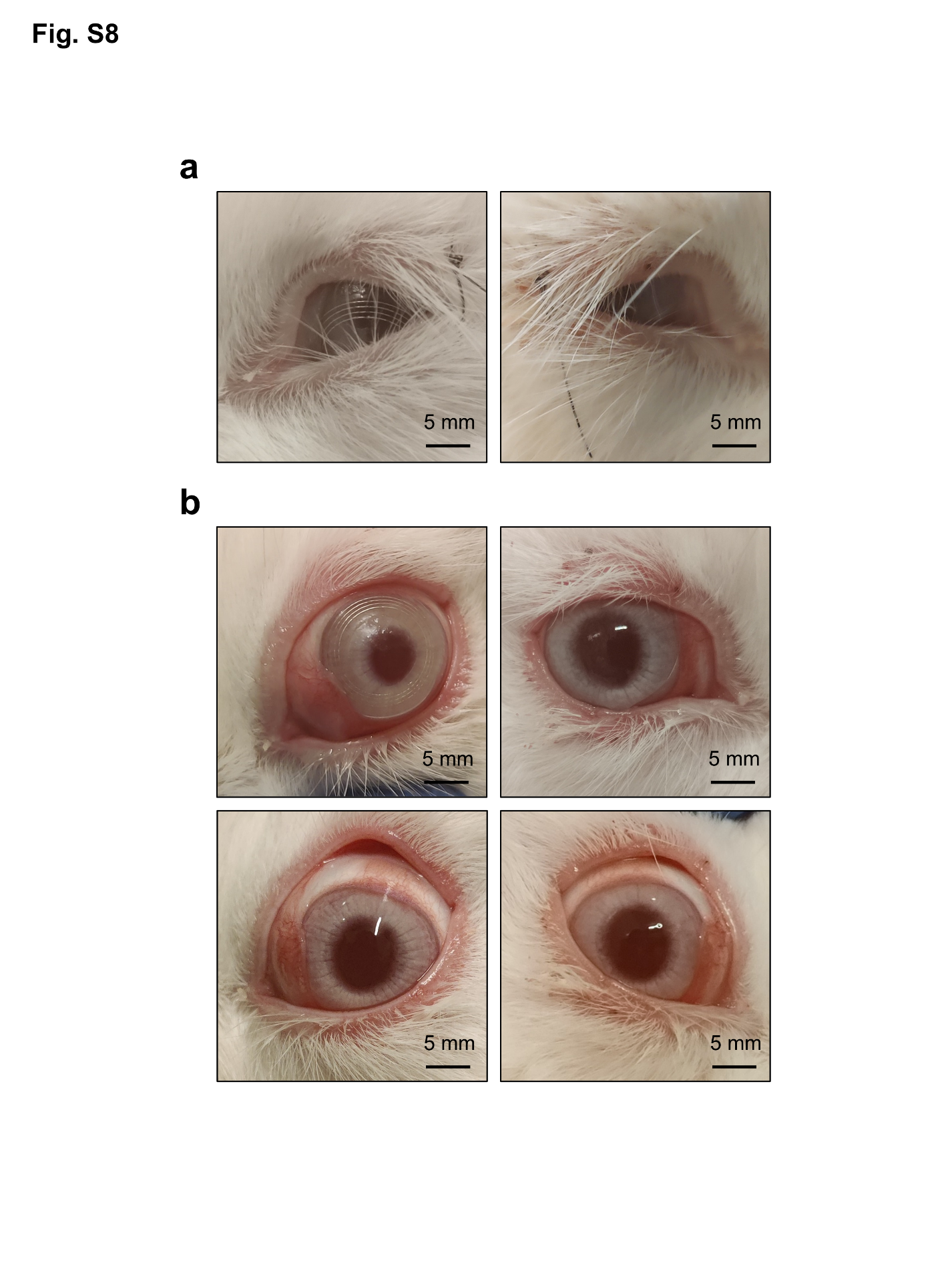


Supplementary Fig. 13: Images of 24-h rabbit biocompatibility test. (a) Sensor (left) and bare lens (right) applied on rabbit eye using partial sutures to ensure maintain position. (b) Post-wear ocular surface images of eyes with the sensor (top left) or bare lens (top right), and after sensor (bottom left) or bare lens (bottom right) removal.


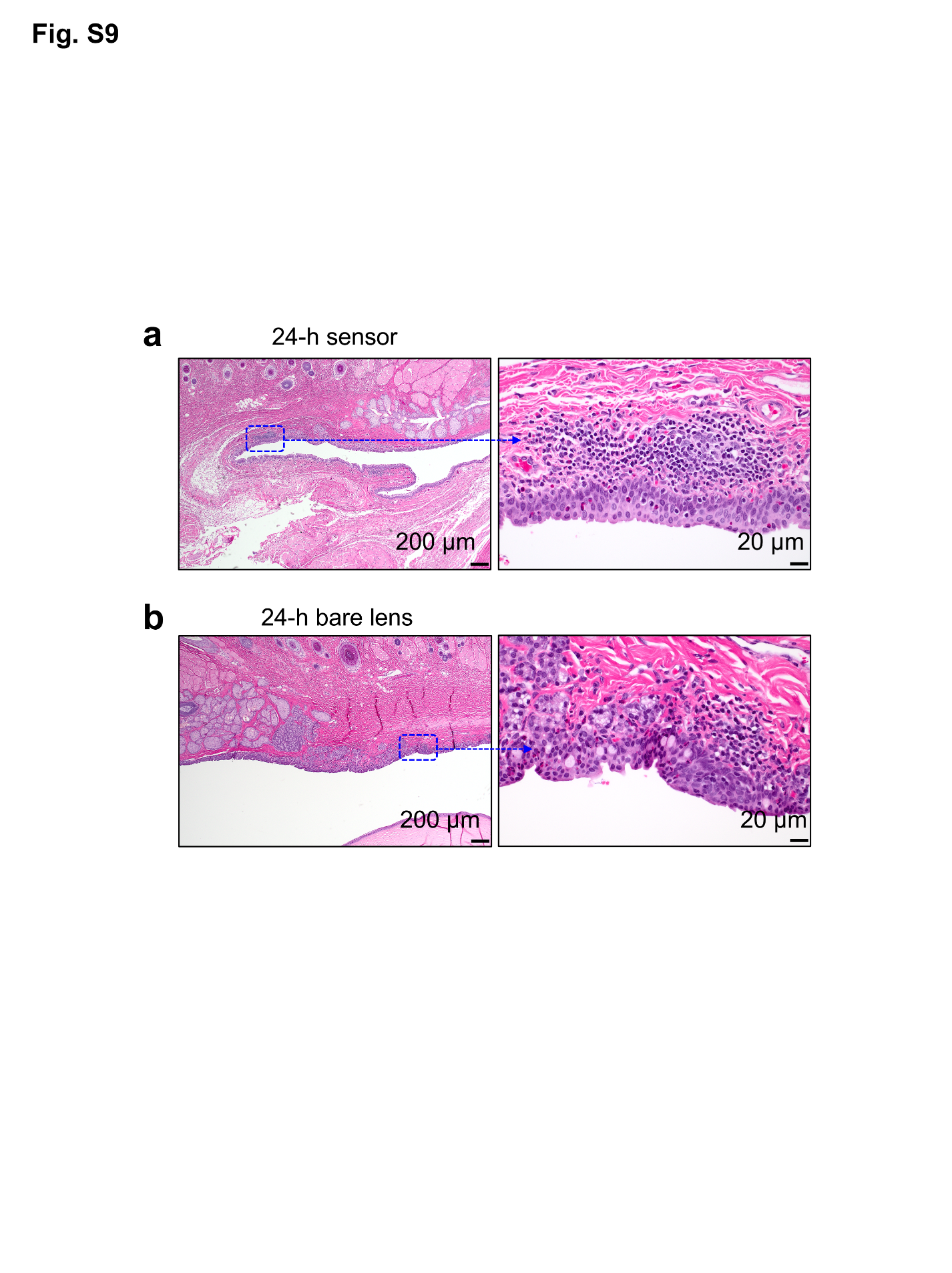


Supplementary Fig. 14: Representative histological images of 24-h rabbit study. (a) Sensor-wearing eye, and (b) control eye with bare lens from the 24-h rabbit study.


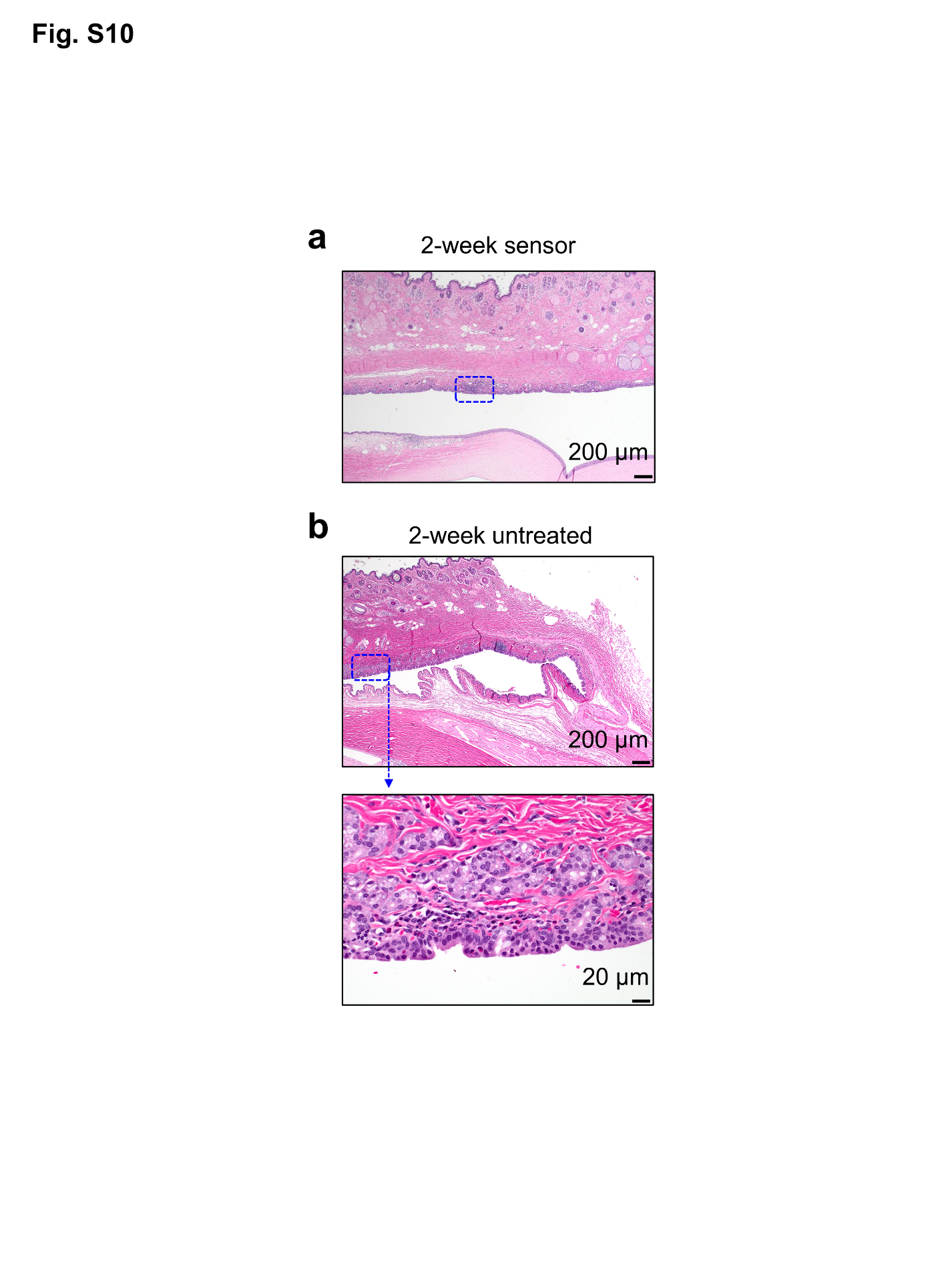


Supplementary Fig. 15: Representative histological images of 2-week rabbit study. (a) Sensor-wearing eye from the 2-week rabbit study. (b) Control untreated eye from the 2-week rabbit study.


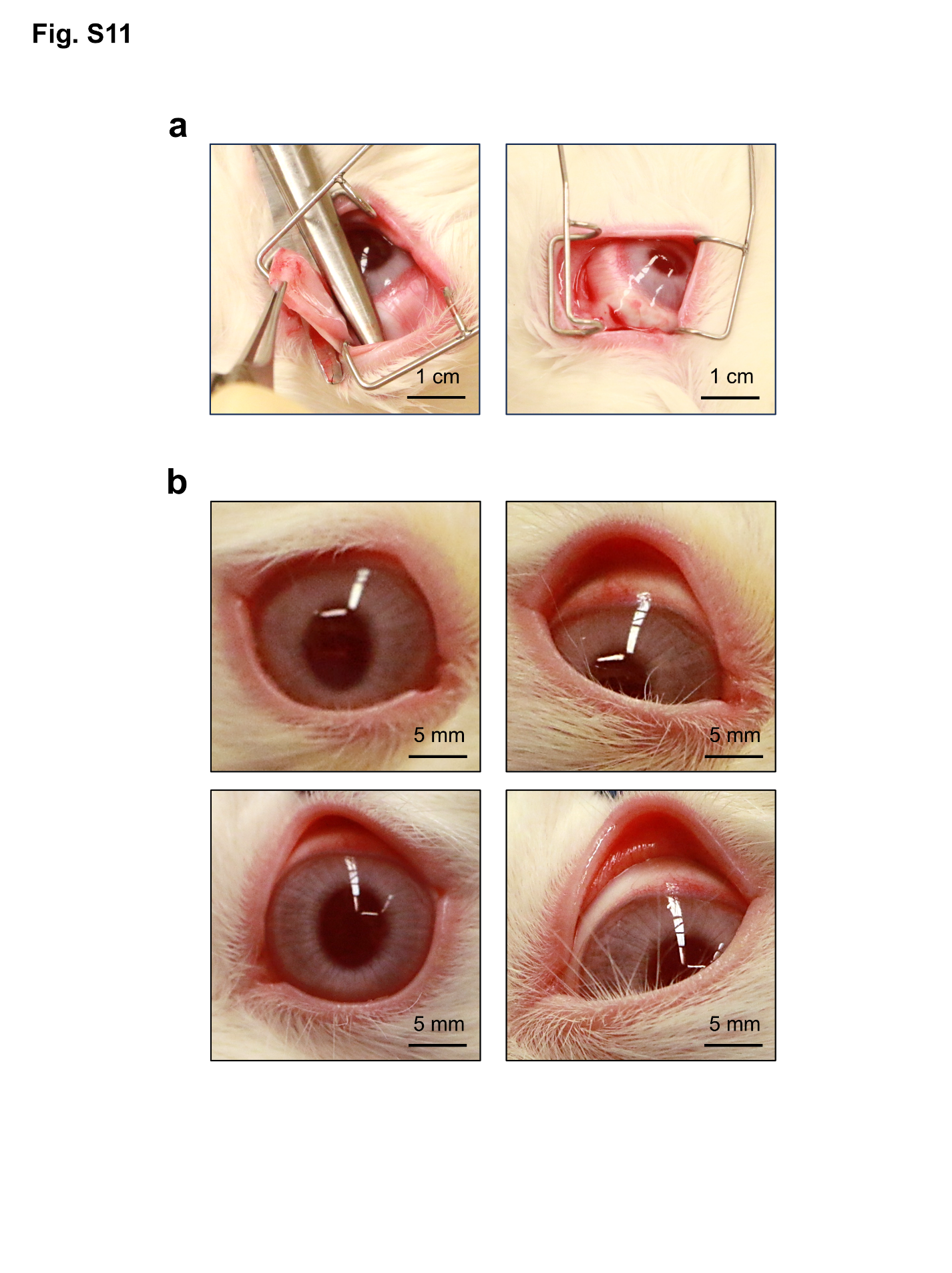


Supplementary Fig. 16: Images of 2-week rabbit biocompatibility test. (a) Photograph of the 3^rd^ eyelid removal process to facilitate sensor retention (left) and the rabbit eye after 3^rd^ eyelid removal (right). (b) Representative post-study images of sensor-treated eye (top) and untreated eye (bottom).


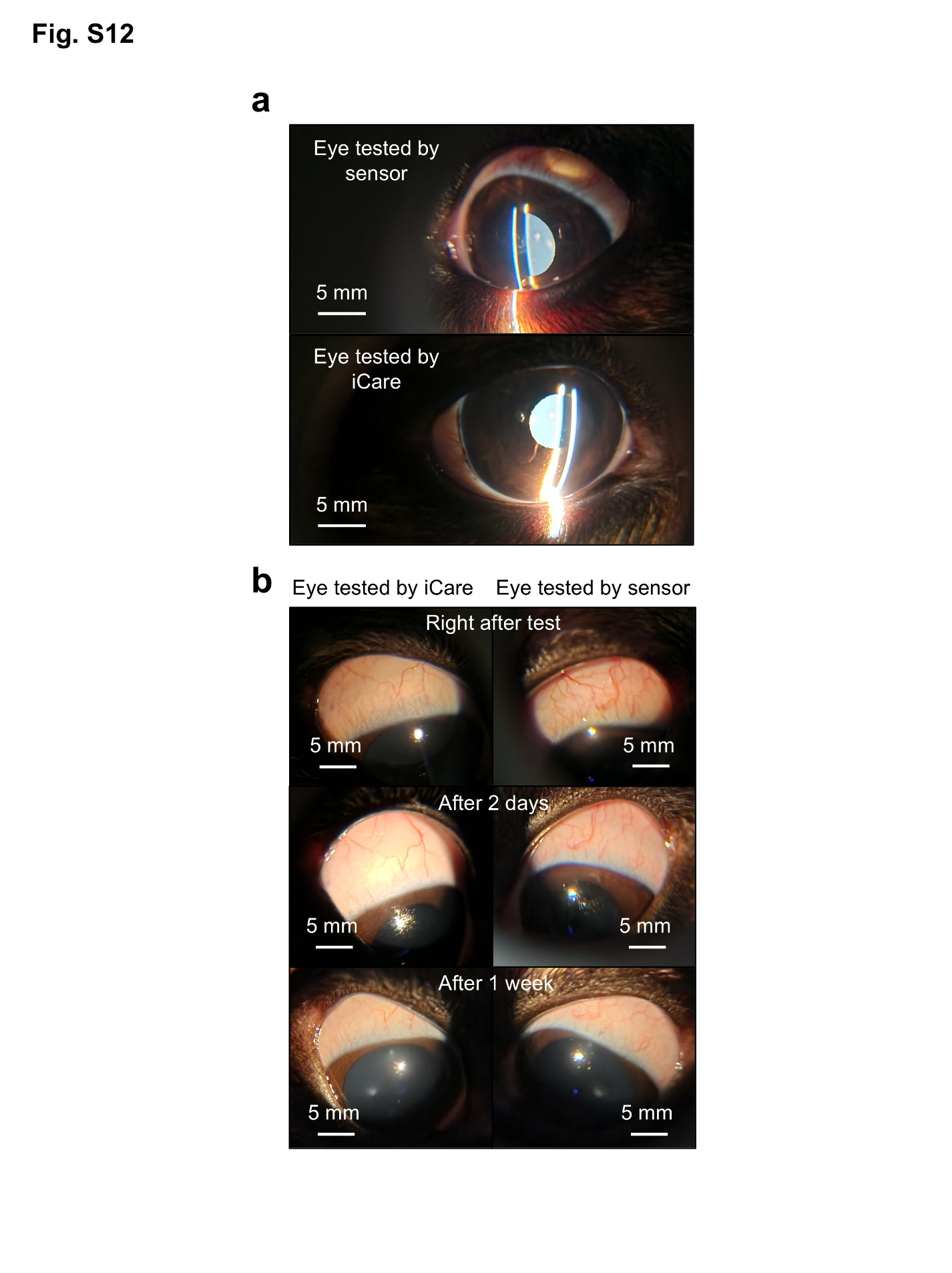


Supplementary Fig. 17: Ocular health evaluation of dog eyes in dog study. (a) Slit lamp examination of dog eyes following sensor wear. (b) Slit lamp images of dog eyes after 24-h monitoring, revealing mild conjunctival hyperemia in both eyes.


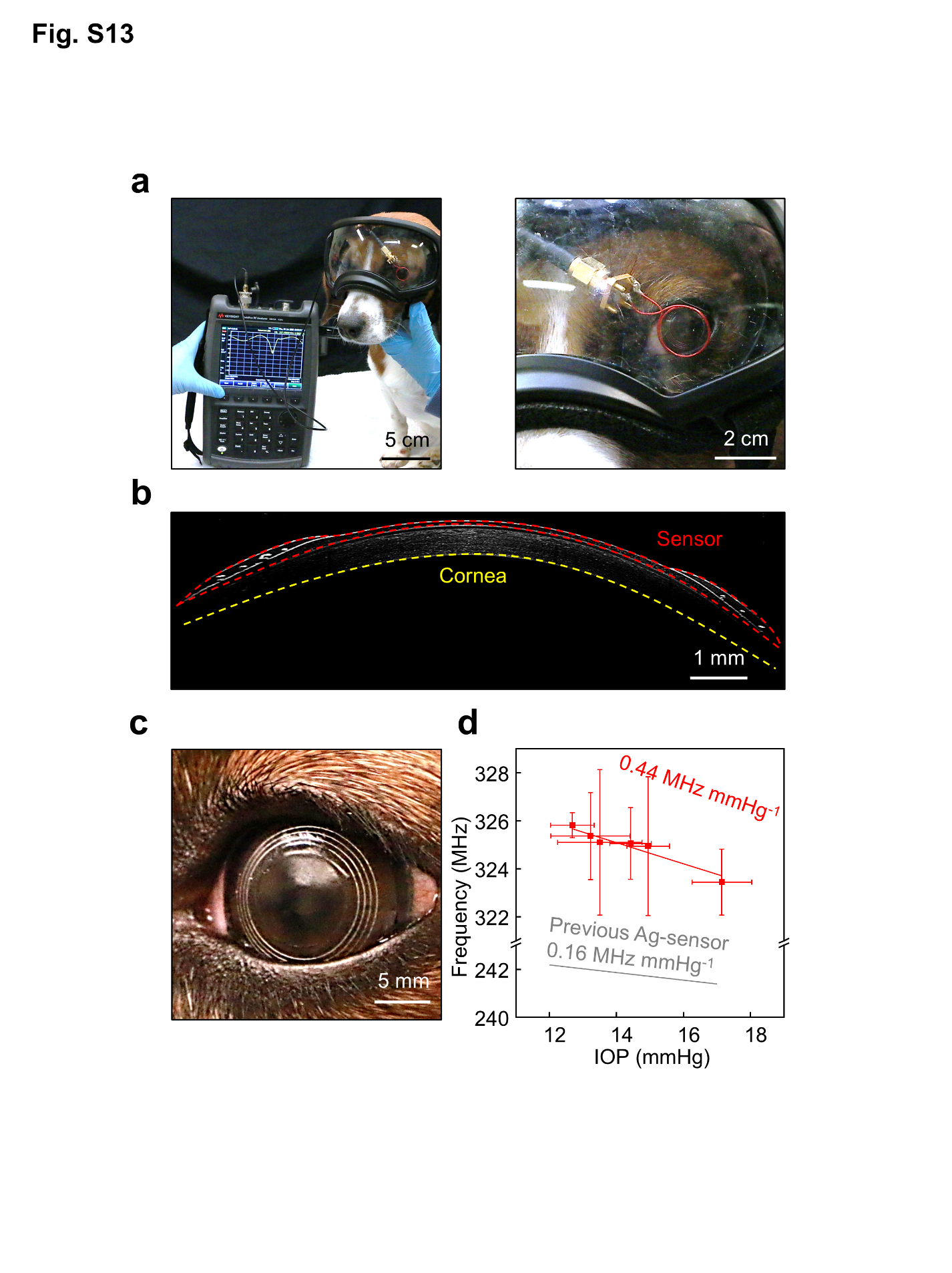


Supplementary Fig. 18: Dog study results. (a) Photograph of the complete experimental setup (left) and the magnified photograph of the dog wearing a coil-integrated custom-fitted goggle (right). (b) AS-OCT of the dog wearing the sensor. (c) Photograph of the dog wearing the sensor. (d) Calibration curve of the sensor, compared with a prior Ag-based sensor^22^.


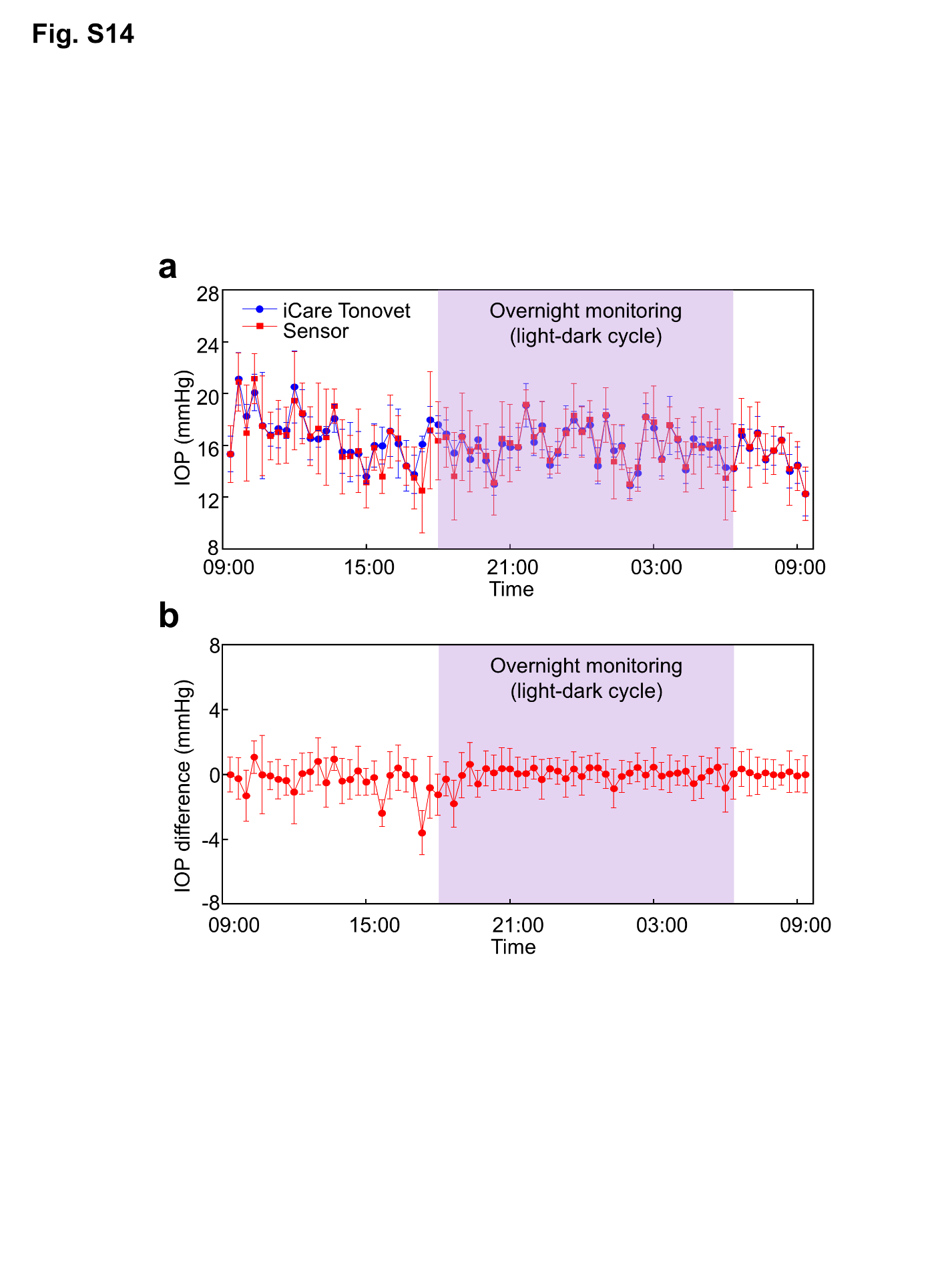


Supplementary Fig. 19: Continuous 24-h study on dogs. (a) 24-h IOP monitoring traces in comparison with the rebound tonometer (iCare Tonovet) readings, with 20 min intervals, and (b) the corresponding difference between IOP measured by iCare and the sensor.


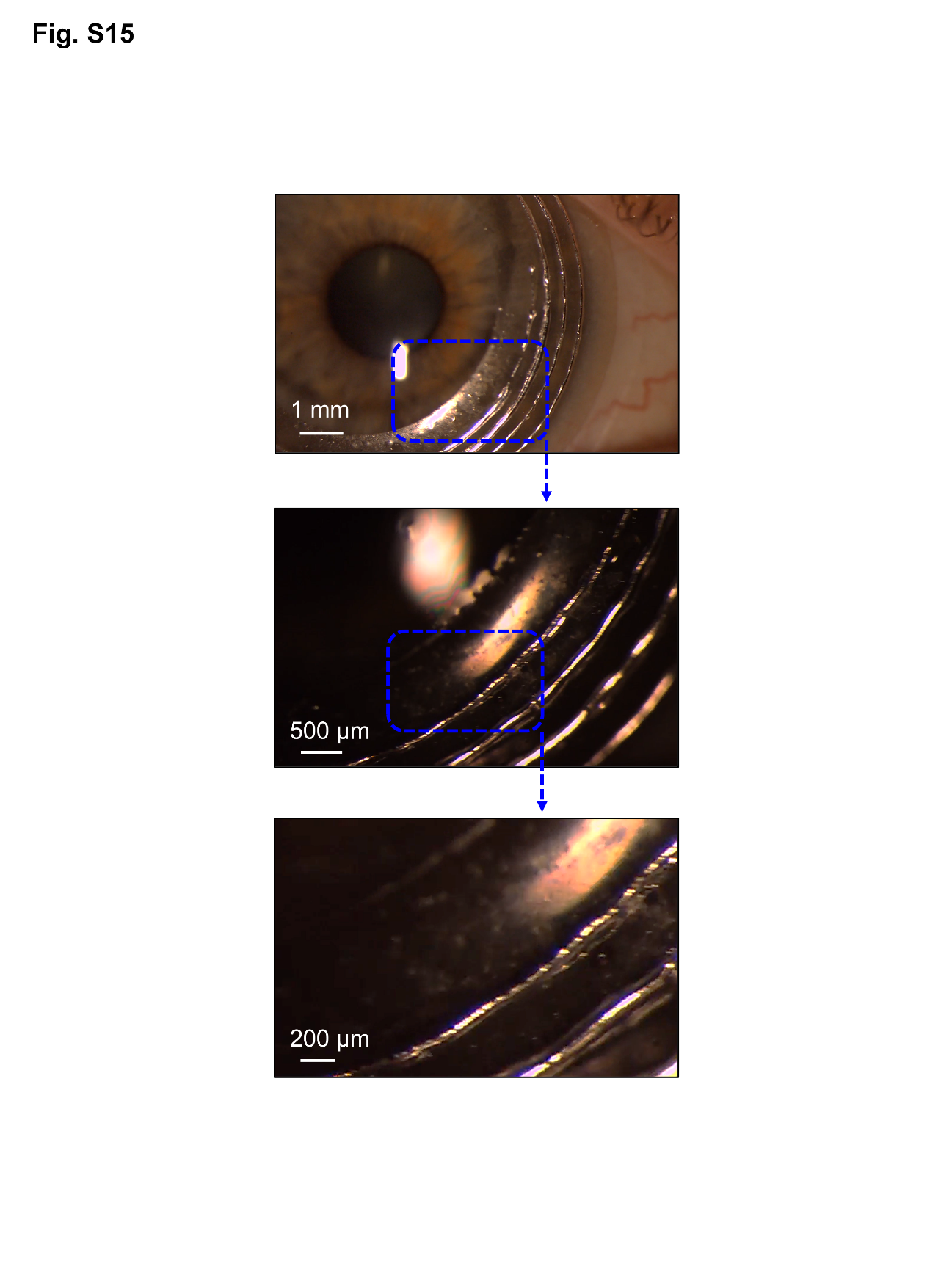


Supplementary Fig. 20: Magnified slit-lamp view of the subject wearing the sensor.


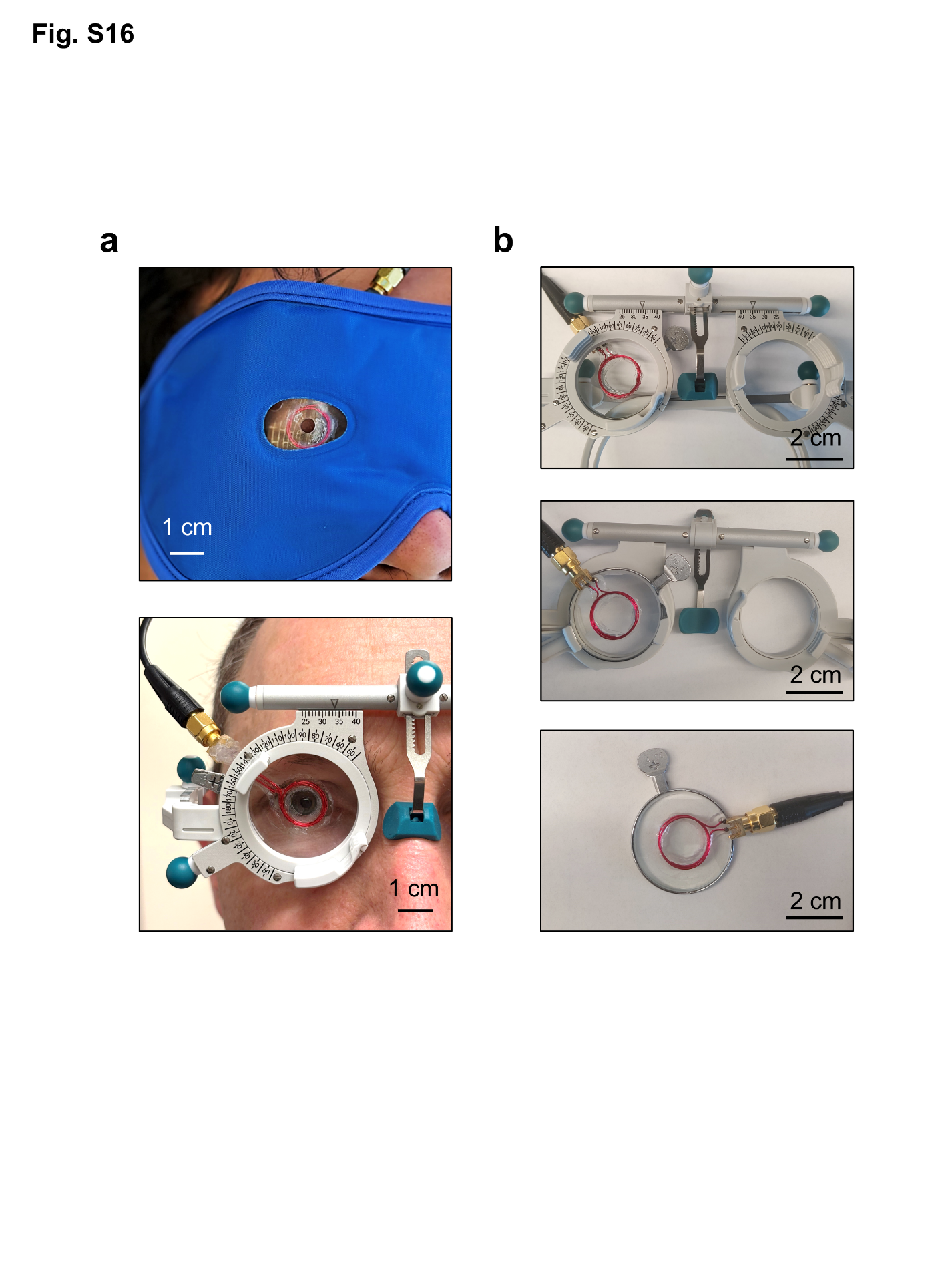


Supplementary Fig. 21: Images for clinical study on healthy subject. (a) Photographs of the representative subject wearing the sleep mask (top) and the trial frame (bottom). (b) Front/back view of the trial frame (top/mid) and the standalone coil-integrated trial glass (bottom).


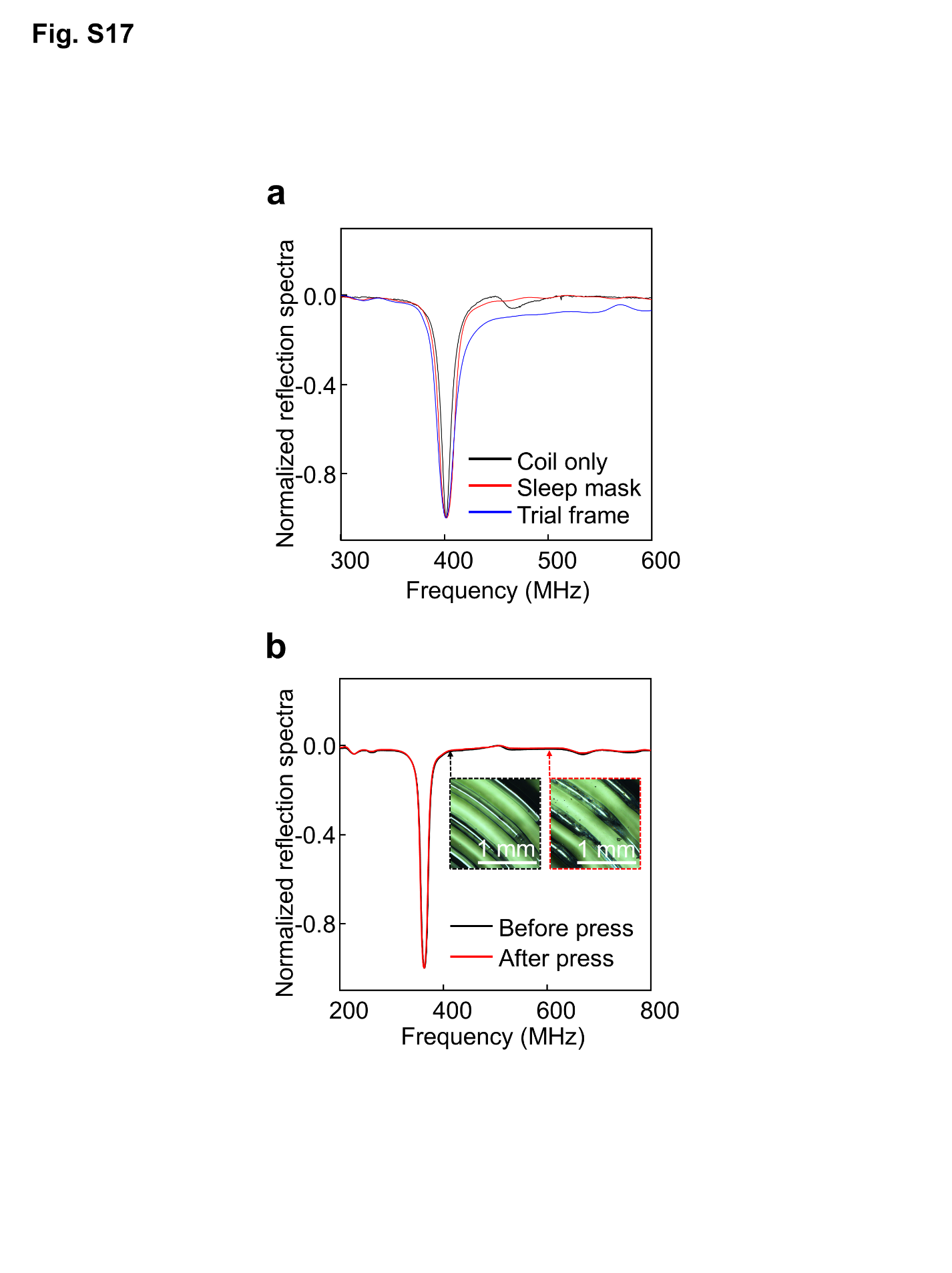


Supplementary Fig. 22: Tolerance of sensing platform and sensor. (a) Benchtop comparison of the standalone reader coil, coil-embedded sleep mask, and coil-integrated trial frame. (b) Applied-pressure testing mimicking eyelid closure, with stable resonant response; pressure was applied by finger, exceeding typical eyelid pressure^32^. Inset shows optical microscopy images of the sensor traces before and after compression.


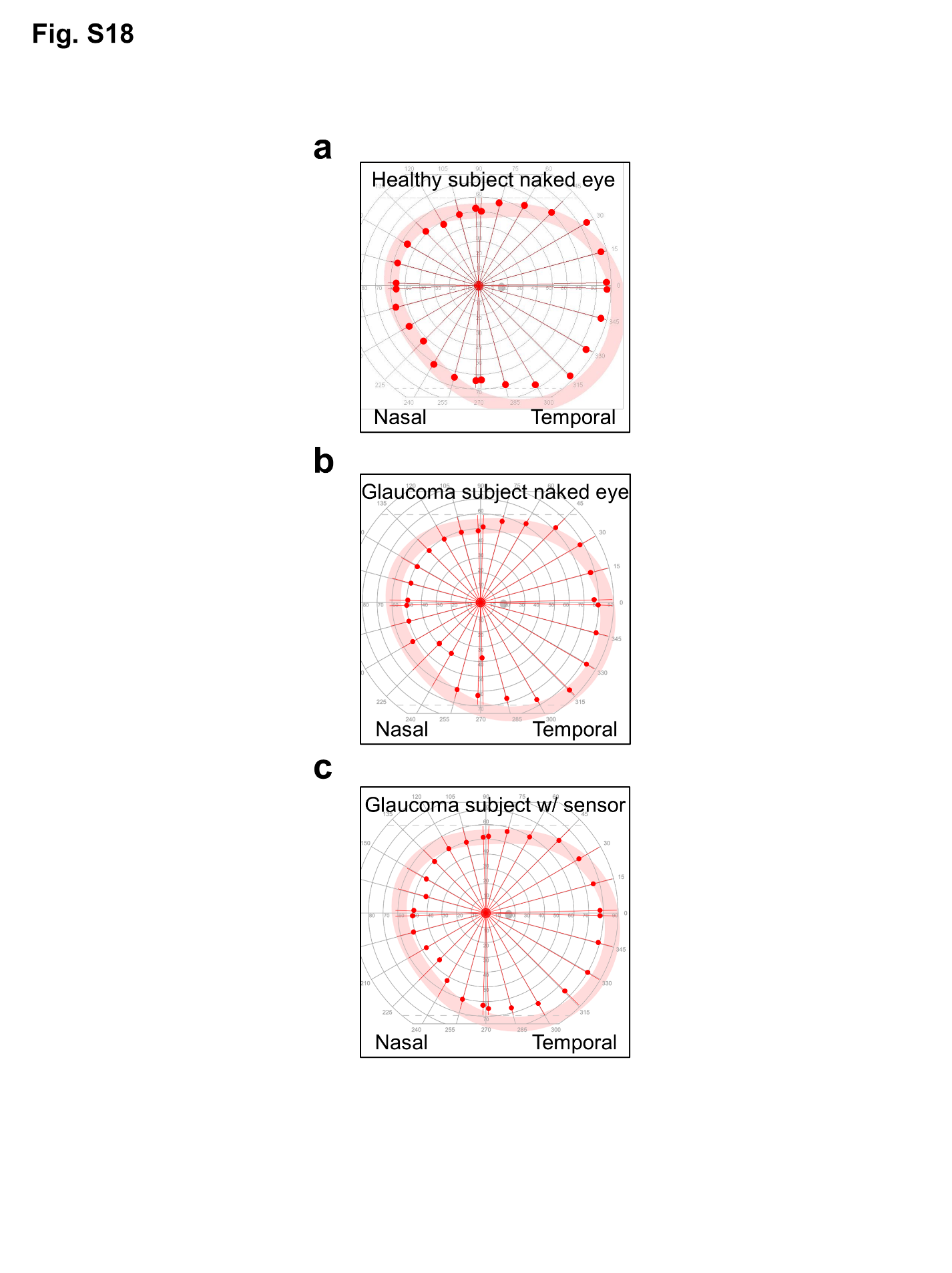


Supplementary Fig. 23: Ocular health evaluation for clinical study. (a) Visual field assessment of the naked-eye in the healthy subject. (b) Visual field assessment of the naked-eye in the glaucoma subject. (c) Visual field assessment of the sensor-wearing eye in the glaucoma subject.


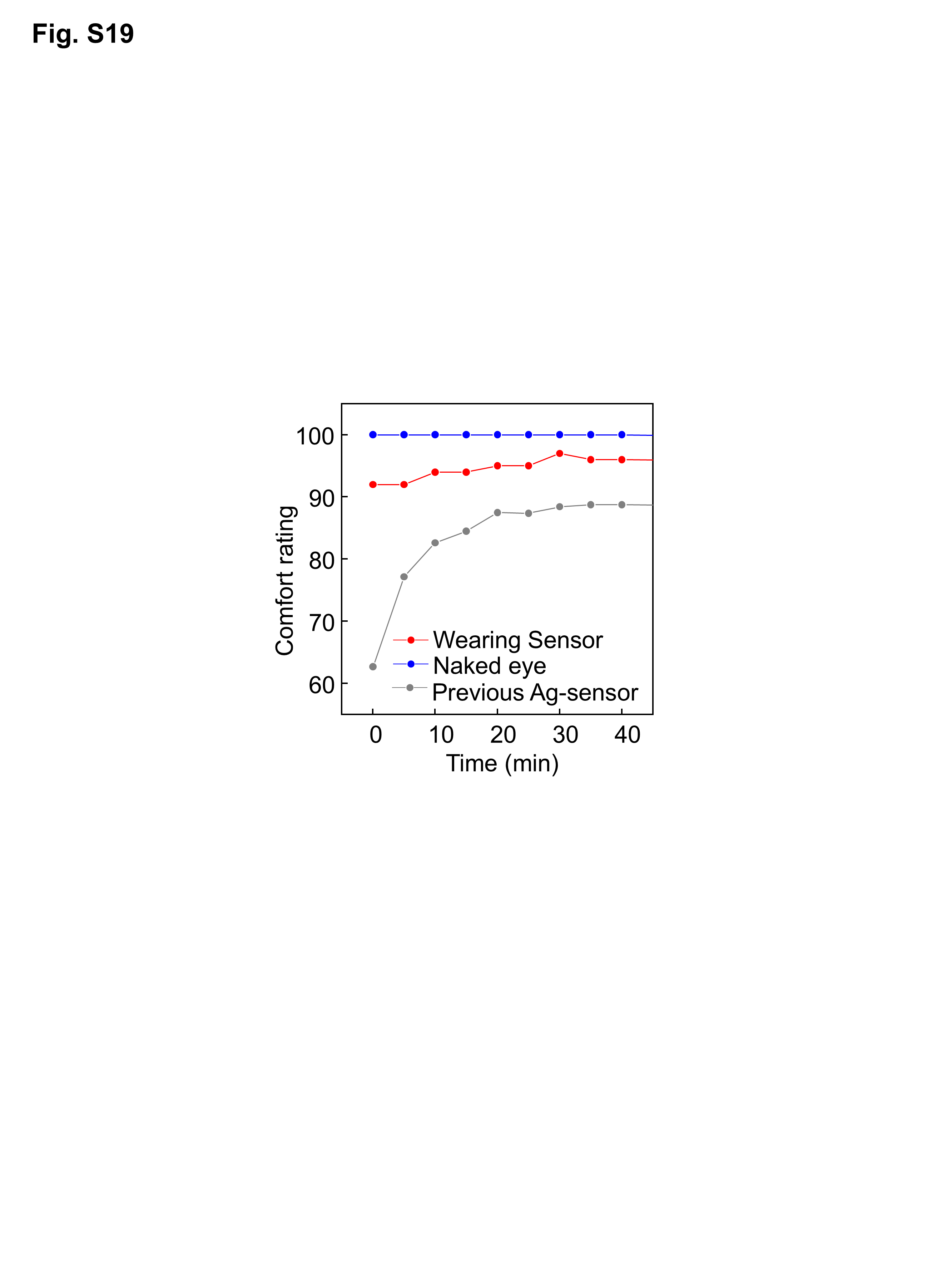


Supplementary Fig. 24: Magnified subjective comfort ratings of the healthy subject during the first 40-min period, compared with a prior Ag-based design^22^.


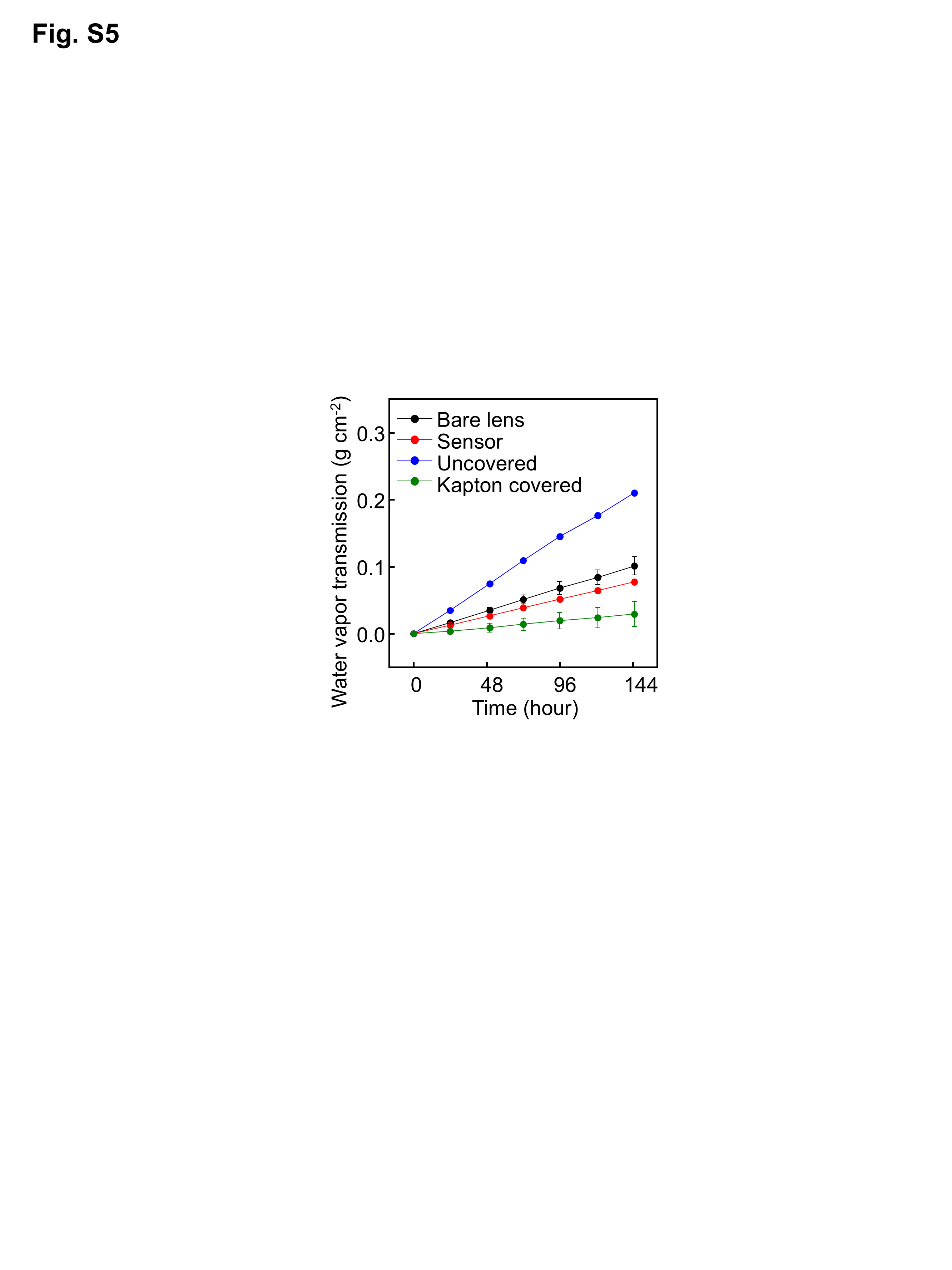


Supplementary Fig. 25: Benchtop water vapor transmission assay of the sensor-integrated contact lens. Cumulative water vapor transmission through the sensor-integrated AirOptix lens, bare AirOptix lens, uncovered vial, and fully Kapton-covered vial over 144 h.


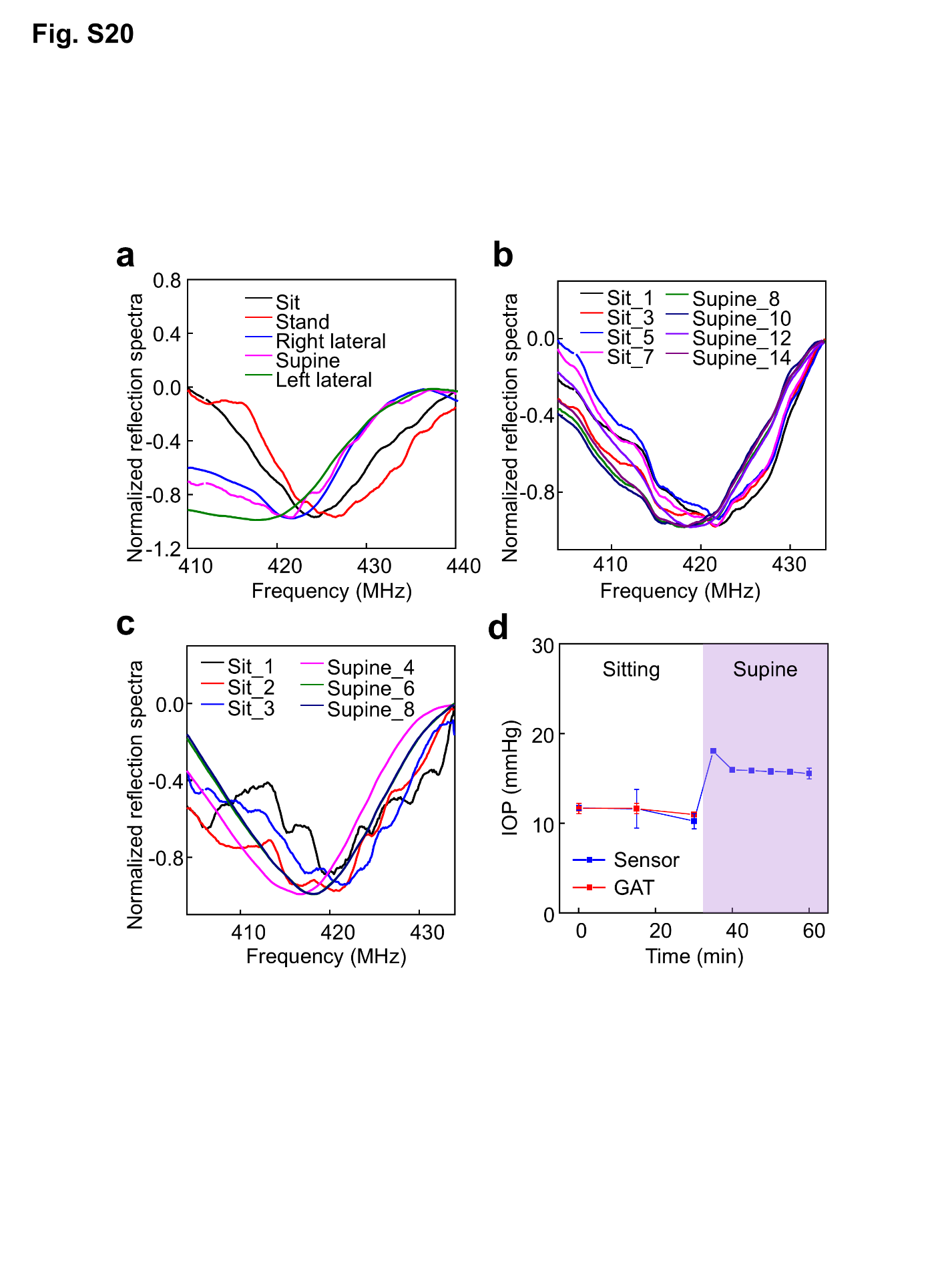


Supplementary Fig. 26: Sensor data for clinical study. (a) Normalized reflection spectra under different postures for calibration in healthy subject. (b) Representative normalized reflection spectra of posture-dependent IOP measurements in the glaucoma subject. Sit_1/3/5/7 refer to sitting posture at time 0/1/2/3 h, and Supine_8/10/12/14 refer to supine posture at time 3.5/4.5/5.5/6.5 h. (c) Representative normalized reflection spectra of posture-dependent IOP measurements in the healthy subject. Sit_1/2/3 refer to sitting posture at time 0/15/30 min, and Supine_4/6/8 refer to supine posture at time 40/50/60 min. (d) Posture-dependent IOP measurement result in the healthy subject, with comparison of GAT readings at sitting posture.


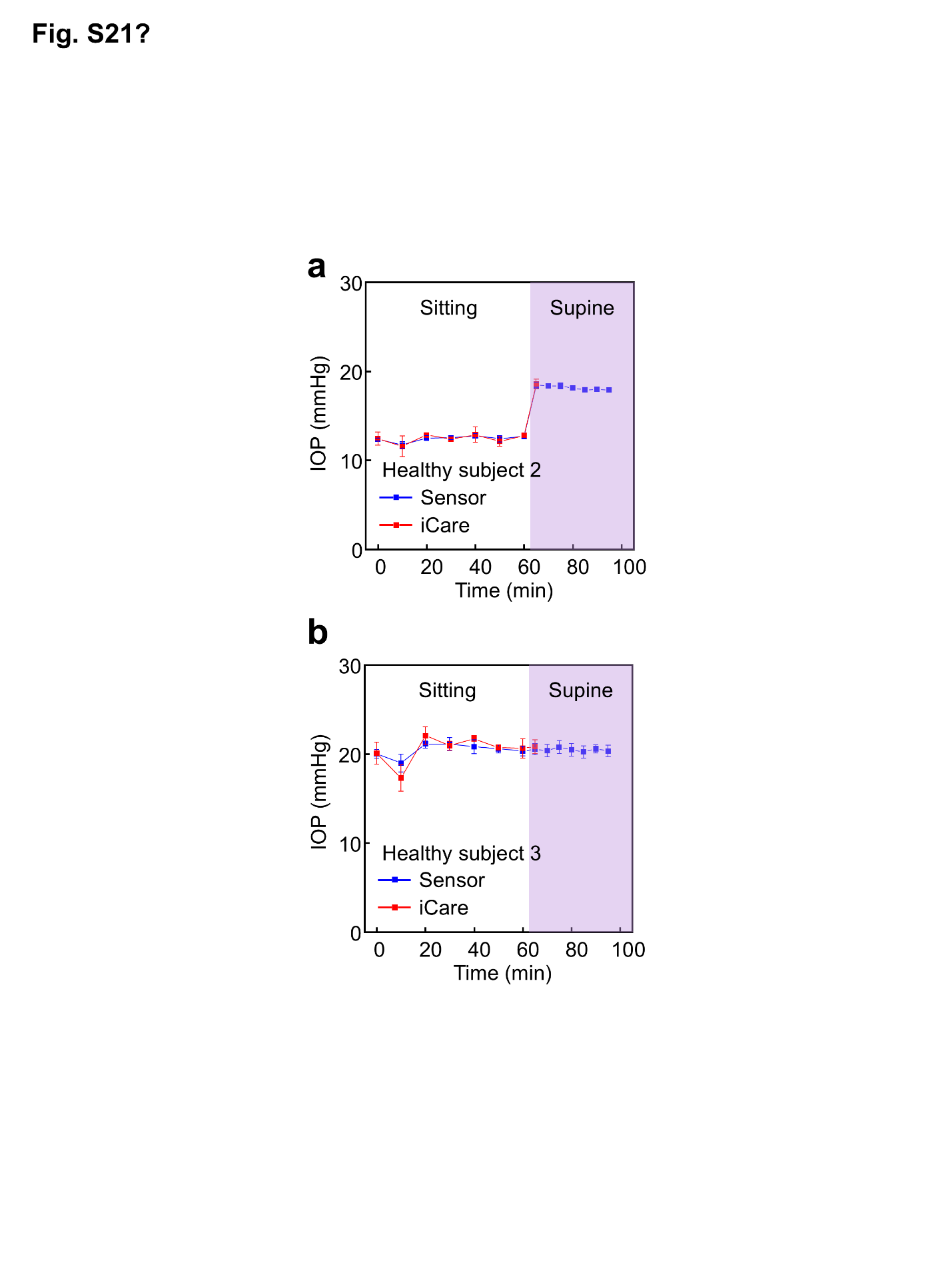


Supplementary Fig. 27: Additional human measurements during sitting-to-supine posture transition testing. (a) Posture-dependent IOP measurement result in healthy subject 2. (b) Posture-dependent IOP measurement result in healthy subject 3.


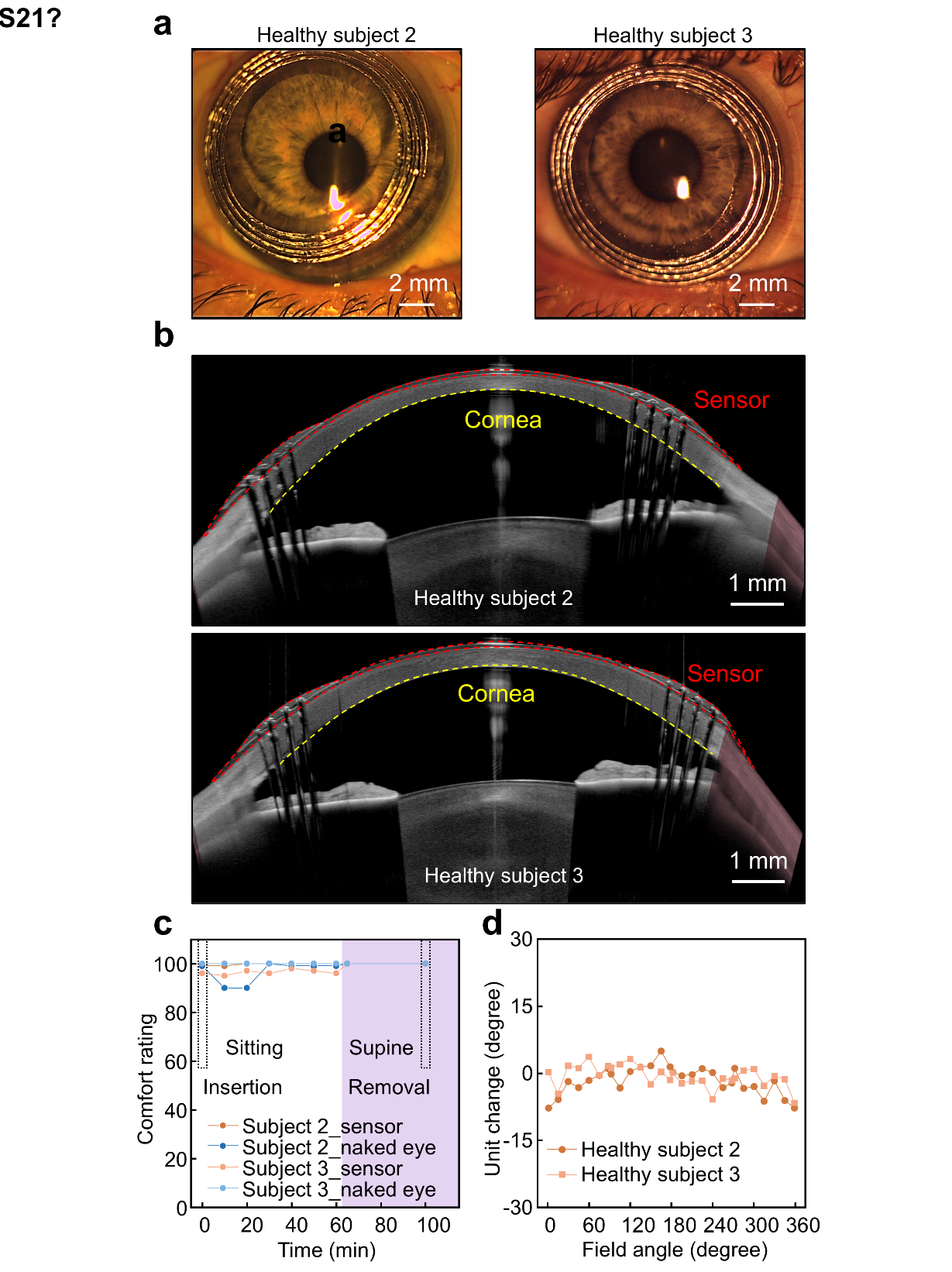


Supplementary Fig. 28: Clinical imaging, comfort assessment, and visual-field evaluation in the two added healthy subjects. (a) Slit-lamp images showing the sensor on the eyes of healthy subjects 2 and 3. (b) AS-OCT images confirming the position of the sensor in both subjects. (c) Comfort ratings recorded during sitting-to-supine measurements for the sensor-wearing eye and the contralateral bare eye. (d) Quantified visual-field changes across field angles.


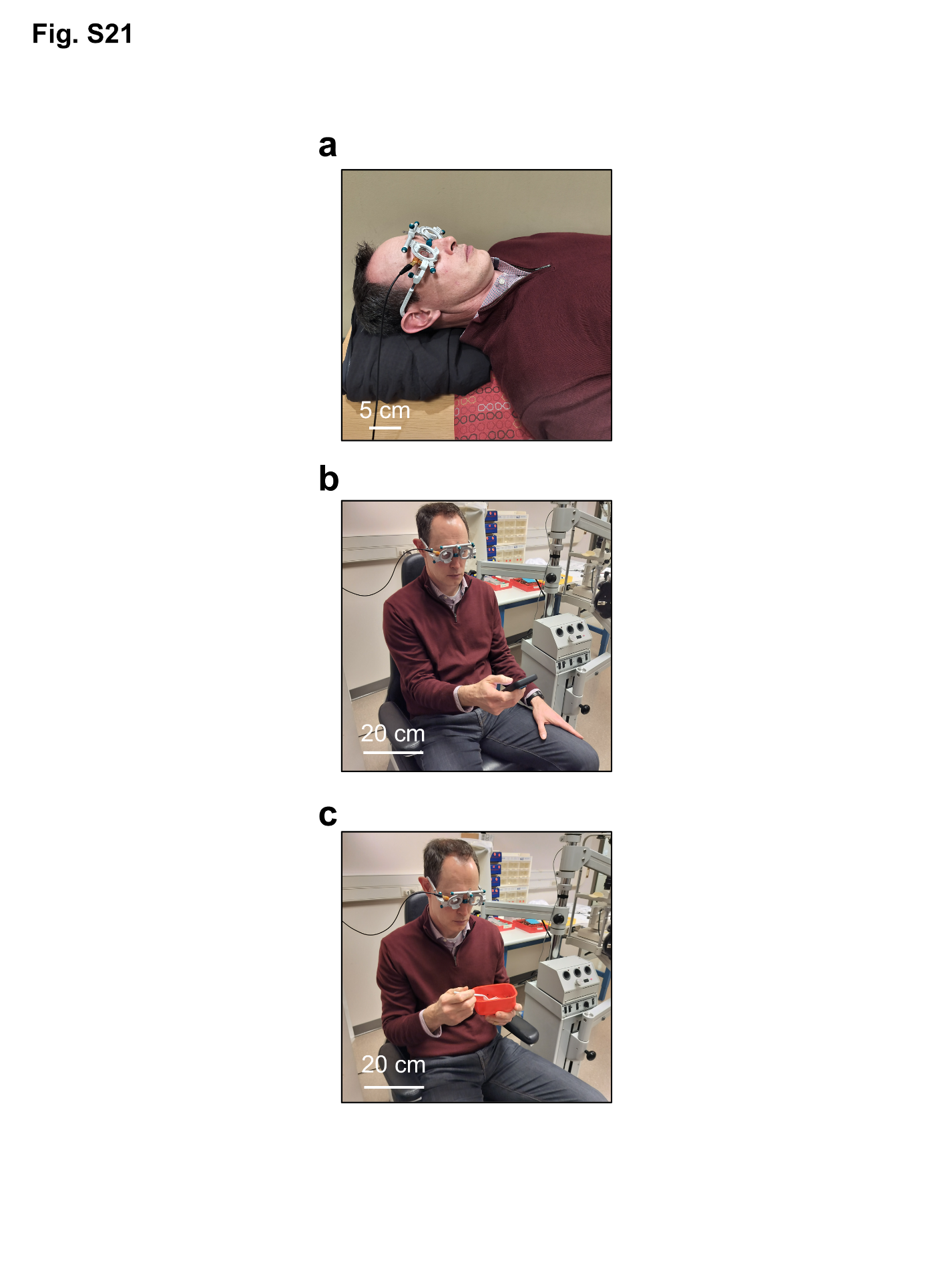


Supplementary Fig. 29: Photographs of the posture-dependent IOP measurements in the glaucoma patient. (a) Photograph of a representative subject wearing the trial frame at supine posture. (b) Photograph of a representative subject using cell phone at sitting posture during experiment. (c) Photograph of a representative subject taking food at sitting posture during experiment.


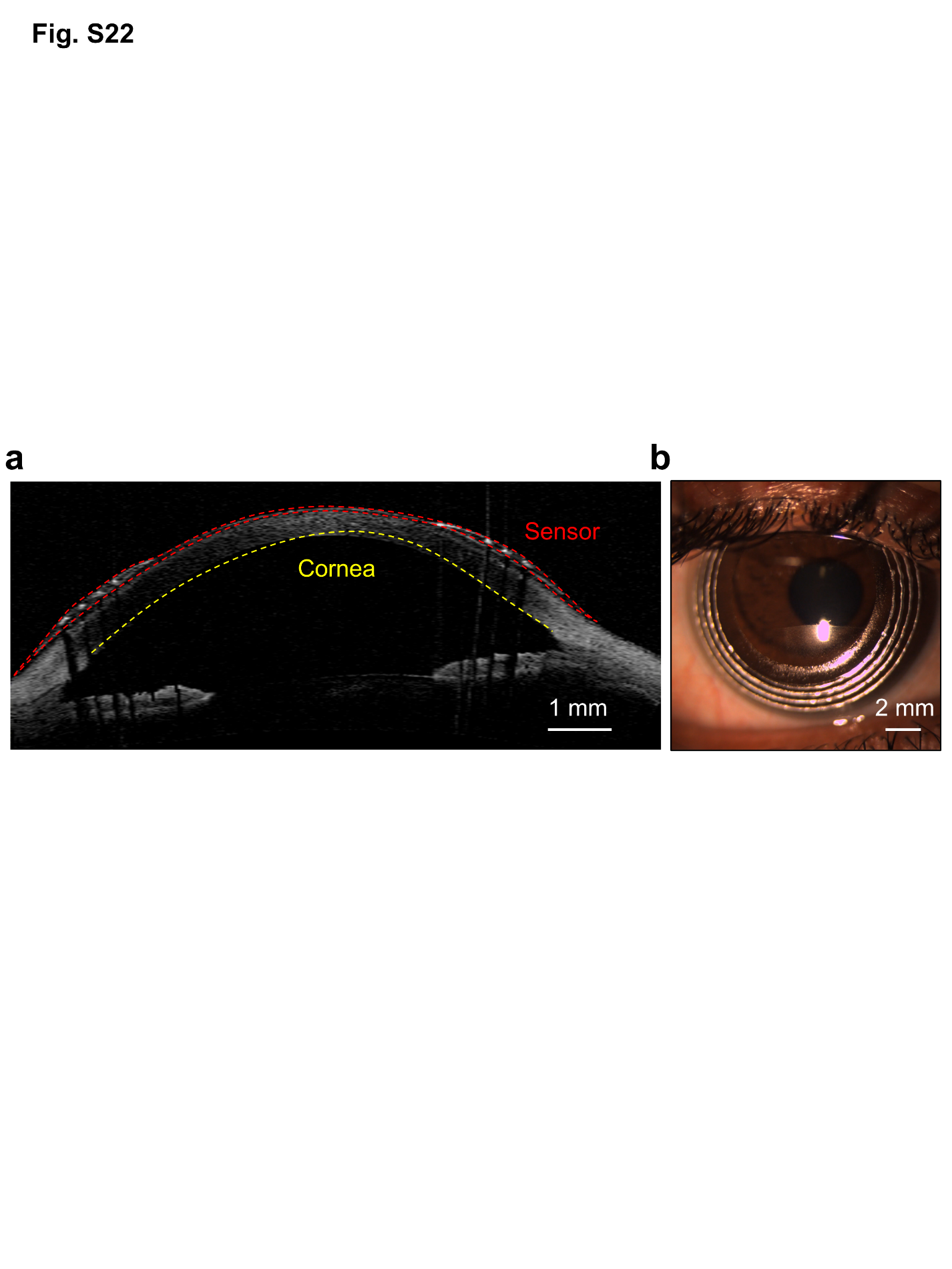


Supplementary Fig. 30: Ocular check of the clinical study in the glaucoma subject. (a) AS-OCT image of the patient wearing the sensor. (b) Slit lamp image of the patient wearing the sensor.


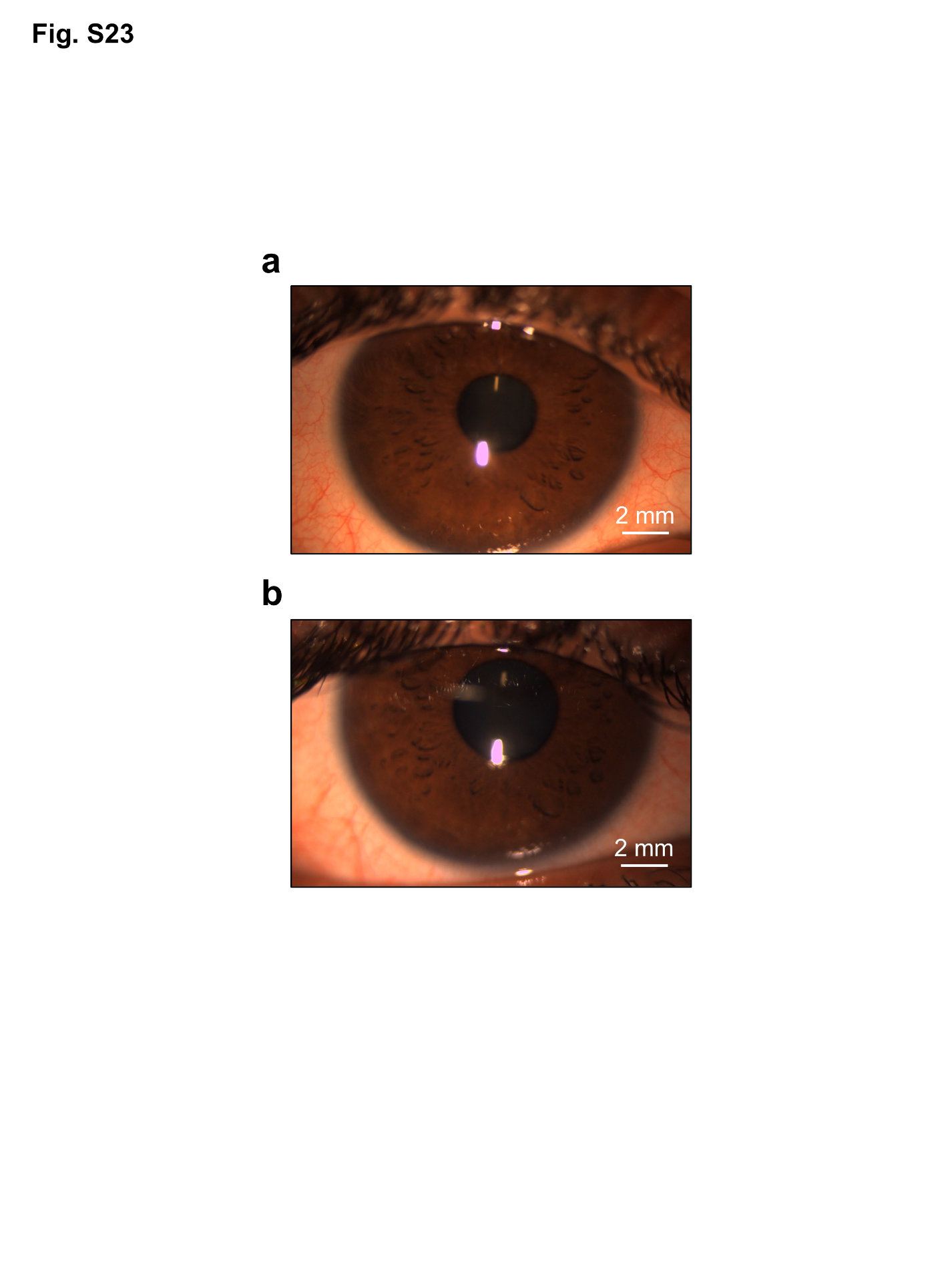


Supplementary Fig. 31: Slit lamp examination in the glaucoma subject. (a) Slit lamp image of right eye before sensor wearing. (b) Slit lamp image of right eye after sensor wearing.

Supplementary Table 1: Comparison of the present soft contact lens sensor with previously reported wearable ocular tonometers based on wireless LC sensing mechanisms.

| Lens substrate | Encapsulation | Dielectric layer | Electrode | Sensitivity (MHz mmHg^-1^) | Original resonant frequency (MHz) | Sensitivity (ppm mmHg^-1^) | Monitoring scenario demonstrated | Ref. |
| --- | --- | --- | --- | --- | --- | --- | --- | --- |
| Commercial | PDMS | Silbione | Ag/SEBS | 0.27 | 241 | 1,121 | 6 h human ambulatory; 24 h in vivo dog | (*22*) |
| Custom PDMS | PDMS | Carbon-doped Ecoflex | LM conductor | 0.131 | 200 | 649 | 30 min human head-down tilt; ex vivo pig eye | (*24*) |
| Custom silicone | Silicone rubber (MED-4286) | Silicone rubber (MED-4286) | LM conductor | 0.70 | 433 | 1,600 | 5 h human lying-position; 6 h in vivo pig | (*2*) |
| Custom silicone | Silicone rubber (MED-4286) | Commercial capacitor | LM conductor | 0.084 | 190 | 442 | In vitro pig eye | (*25*) |
| Custom PDMS | PDMS | Parylene C | Au | 0.0351 | 490 | 72 | Ex vivo dog eye | (*26*) |
| Custom pHEMA hydrogel | PET | Ecoflex | Cu | 0.109 | 99 | 1,100 | In vitro pig eye; glasses-integrated remote readout | (*27*) |
| Custom PDMS | PDMS | Air | Cu/Ni/Au on polyimide | 1.28 | 3,800 | 337 | 3 h in vivo rabbit sensing and therapeutic test; ex vivo pig eye | (*28*) |
| Commercial | Ecoflex & PDMS | Silbione | LM conductor | 1.08 | 425 | 2,540 | >6 h human sitting-to-supine with a closed-eye phase mimicking sleep; 24 h in vivo dog | This work |

Supplementary Table 2: Comparison of sensors on different brands of soft contact lenses.

| Brand name | Young’s modulus (MPa) | Water content | Center thickness (mm) | Responsivity  (MHz mmHg^-1^) | *R*^2^ | Sensitivity  (ppm mmHg^-1^) |
| --- | --- | --- | --- | --- | --- | --- |
| AirOptix | 1.90 | 24% | 0.080 | -0.163 ± 0.009 | 0.977 | -639 ± 35 |
|  |  |  |  | -0.125 ± 0.009 | 0.965 | -470 ± 34 |
|  |  |  |  | -0.096 ± 0.008 | 0.955 | -349 ± 29 |
| Biofinity | 0.80 | 48% | 0.080 | -0.215 ± 0.023 | 0.924 | -766 ± 82 |
|  |  |  |  | -0.151 ± 0.022 | 0.867 | -580 ± 85 |
|  |  |  |  | -0.237 ± 0.019 | 0.958 | -662 ± 53 |
| Oasys (Acuvue Oasys) | 0.49 | 38% | 0.085 | -0.014 ± 0.007 | 0.272 | -64 ± 32 |
|  |  |  |  | 0.089 ± 0.016 | 0.817 | 341 ± 60 |
|  |  |  |  | 0.069 ± 0.027 | 0.442 | 258 ± 101 |

Supplementary Table 3: Evaluation of histopathologic inflammation severity in rabbit cornea and conjunctiva.

| Rabbit Number | Study type | Inflammation level ^[3]^ | | |
| --- | --- | --- | --- | --- |
|  |  | Eye-sensor | Eye-bare lens | Eye-untreated |
| 1 | 24-h ^[1]^ | 1 | 0.75 | - |
| 2 | 24-h ^[1]^ | 0.75 | 1 | - |
| 3 | 24-h ^[1]^ | 0.5 | 0.75 | - |
| 4 | 2-week ^[2]^ | 0.75 | - | 0.5 |
| 5 | 2-week ^[2]^ | 0.75 | - | 0.75 |
| 6 | 2-week ^[2]^ | 0.5 | - | 0.75 |
| [1] One eye of the rabbit was fitted with the sensor for 24 hours, while the other eye was fitted with a bare lens.  [2] One eye of the rabbit was fitted with the sensor for 8 hours per day over a 2-week period, while the other eye remained untreated.  [3] Quantified grades of inflammation: Minimal – 0.5, Mild – 1, Moderate – 2, Severe – 3 | | | | |

Supplementary Table 4: Approximate constituent-layer thicknesses of the integrated sensor and simplified area-weighted estimation of effective modulus.

| Material | Width (μm) | Repetition factor | Thickness (μm) | Modulus (MPa) |
| --- | --- | --- | --- | --- |
| PDMS | 400 | 4 | 50 ^[1]^ | 0.70 ^[2]^ |
| Silbione | 400 | 4 | 20 ^[1]^ | 0.010 ^[2]^ |
| Ecoflex | 3,400 | 1 | 75 ^[1]^ | 0.069 ^[2]^ |
| Effective modulus (area-weighted) ^[3]^ | - | - | - | 0.20 |
| [1] Layer thicknesses were approximately estimated from a representative cross-sectional SEM image of the integrated sensor region.  [2] Material moduli were obtained from manufacturer datasheets.  [3] A simplified area-weighted effective modulus was calculated based on the thickness and modulus of each constituent layer, and the liquid-metal conductor was not assigned a solid elastic modulus and was therefore excluded from the area-weighted calculation. The resulting value should be interpreted as an approximate compliance-related estimate of the multilayer sensor structure rather than a full mechanical model of the integrated sensor-lens composite. | | | | |

Supplementary Table 5: Summary of stable lens-mounted bench-top measurements used for practical-resolution estimation.

| Trace | Number of points | Mean resonant frequency (MHz) | Standard deviation (MHz) | Practical resolution (mmHg) |
| --- | --- | --- | --- | --- |
| 1 | 150 | 613.52 | 0.244 | 0.68 |
| 2 | 150 | 613.65 | 0.306 | 0.85 |
| 3 | 150 | 613.73 | 0.302 | 0.84 |
| Pooled stable traces | 450 | 613.63 | 0.298 | 0.83 |
| [1] The practical resolution was estimated as $\boldsymbol{3}\boldsymbol{\sigma}_{\boldsymbol{f}}\boldsymbol{/\mid}\boldsymbol{S}\boldsymbol{\mid}$, where $\boldsymbol{\sigma}_{\boldsymbol{f}}$is the standard deviation of the resonant frequency fluctuation and *S* is the calibration sensitivity (1.08 MHz mmHg^-1^) obtained from clinical study. | | | | |

Supplementary Video 1: Application and removal of the soft contact lens sensor in a dog model.

This movie shows the placement and removal of the sensor on a dog eye using procedures analogous to commercial contact lens handling.

Supplementary Video 2: Continuous IOP measurement in a dog model using the soft contact lens sensor.

This movie demonstrates real-time IOP monitoring in a dog eye using the sensor during in vivo testing.

Supplementary Video 3: Reference IOP measurement in a dog model using rebound tonometry.

This movie shows IOP measurement in the contralateral dog eye using a commercial iCare Tonovet rebound tonometer, serving as a reference during sensor calibration.

Supplementary Video 4: Application and removal of the soft contact lens sensor in a human subject.

This movie demonstrates self-application and removal of the sensor by a human subject, following standard soft contact lens handling procedures.

Supplementary Video 5: Posture-dependent IOP calibration in a human subject using the soft contact lens sensor.

This movie shows posture-dependent IOP calibration in a human subject using the sensor across sitting, standing, lateral, and supine positions.

Supplementary Video 6: Posture-dependent IOP calibration in a human subject using rebound tonometry.

This movie shows corresponding iCare Home rebound tonometry measurements obtained from the contralateral eye across different postures during human IOP calibration.

Supplementary Video 7: Evaluation of motion robustness of the soft contact lens sensor in a human subject.

This movie shows IOP measurement in a human subject using the sensor across different motion conditions, including horizontal eye movement, vertical eye movement, horizontal head movement, vertical head movement, and rapid blinking.

Supplementary Video 8: Sensor application and removal in the two added healthy subjects.

This movie shows application and removal of the sensor-integrated soft contact lens in healthy subjects 2 and 3 using standard soft contact lens handling procedures.

Supplementary Video 9: IOP monitoring during common daily activities in a human subject.

This movie shows continuous sensor-based IOP monitoring while the subject performs typical daily activities, including talking, eating, using cell phone, and napping/sleeping.
